# Ancestry & molecular evolutionary analyses of heat shock protein 47 kDa (HSP47/SERPINH1)

**DOI:** 10.1038/s41598-017-10740-0

**Published:** 2017-09-04

**Authors:** Abhishek Kumar, Anita Bhandari, Sandeep J. Sarde, Chandan Goswami

**Affiliations:** 1 Department of Genetics & Molecular Biology in Botany, Institute of Botany, Christian-Albrechts-University at Kiel, Kiel, Germany; 2 Institute for Cardiogenetics, University of Lübeck, Lübeck, Germany; 3 Laboratory of Entomology, Department of Plant Sciences, Wageningen University, Wageningen, Netherlands; 4 National Institute of Science Education and Research, Bhubaneswar, Orissa India; 5 Present Address: Division of Molecular Genetic Epidemiology German Cancer Research Center, Heidelberg, Germany

## Abstract

HSP47/SERPINH1 is key-regulator for collagen biosynthesis and its structural assembly. To date, there is no comprehensive study on the phylogenetic history of HSP47. Herein we illustrate the evolutionary history of HSP47/SERPINH1 along with sequence, structural and syntenic traits for HSP47/SERPINH1. We have identified ancestral HSP47/SERPINH1 locus in Japanese lamprey (*Lethenteron japonicum*). This gene remains on the same or similar locus for ~500 million years (MY), but chromosomal duplication was observed in ray-finned fishes, leading into three sets of three sets (I-III) of HSP47/SERPINH1. Two novel introns were inserted at the positions 36b and 102b in the first exon of only HSP47_1 gene from the selected ray-finned fishes. On the evolutionary time scale, the events of HSP47 duplications took placed between 416–360 MY ago (MYA) while intron insertion dates back to 231–190 MYA after early divergence of ray-finned fishes.

## Introduction

Heat shock protein 47 (HSP47/SERPINH1) operates as a client-specific chaperone for collagen and plays a vital role in collagen maturation and the consequent embryonic development. It is a non-inhibitory serpin, which belongs to the clade H and group V6 based on the clade and indel based group-wise classification systems of vertebrate serpins, respectively^1^. This serpin is an ER-resident, collagen-specific chaperone and plays a key role in collagen biosynthesis and its structural assembly^2^.

A typical three-dimensional structure of serpins possesses three β-sheets (sA-sC) and 8–9 α-helices (hA-hI)^3^. The physiological peculiarity of serpins is the reactive center loop (RCL), which is composed of ~17–20 residues and the RCL region gimmicks proteases for their subtract and proteases bind and cleave between the active sites P1 and P1’^3^ in the RCL region and ultimately inactivated along with serpins. Molecular diversities of different serpins are created by the addition of extra sequences at the terminal end of this core domain structures and also by mutational changes in their RCL regions. Over last five decades, HSP47/SERPINH1 has been extensively characterized by biochemical and biophysical methods to demonstrate its roles in the collagen biosynthesis. However, a comprehensive molecular phylogenetic analysis of HSP47/SERPINH1 has not been focused. This is because of two facts that (a) the difficulties associated with the reconstruction of phylogenetic relationships among different animals and presence of several paralogs in various animals^4^ and (b) lack of sufficient vertebrate genomes, previously. Hence, an investigation on molecular phylogenetic aspects of HSP47/SERPINH1 is warranted. Herein, we elucidated the detailed molecular phylogeny of HSP47/SERPINH1 genes by combining protein sequence, gene structures and genomic organization from 61 vertebrate genomes.

## Results

### Overview of HSP47/SERPINH1 repository in vertebrate genomes

**Figure 1:**
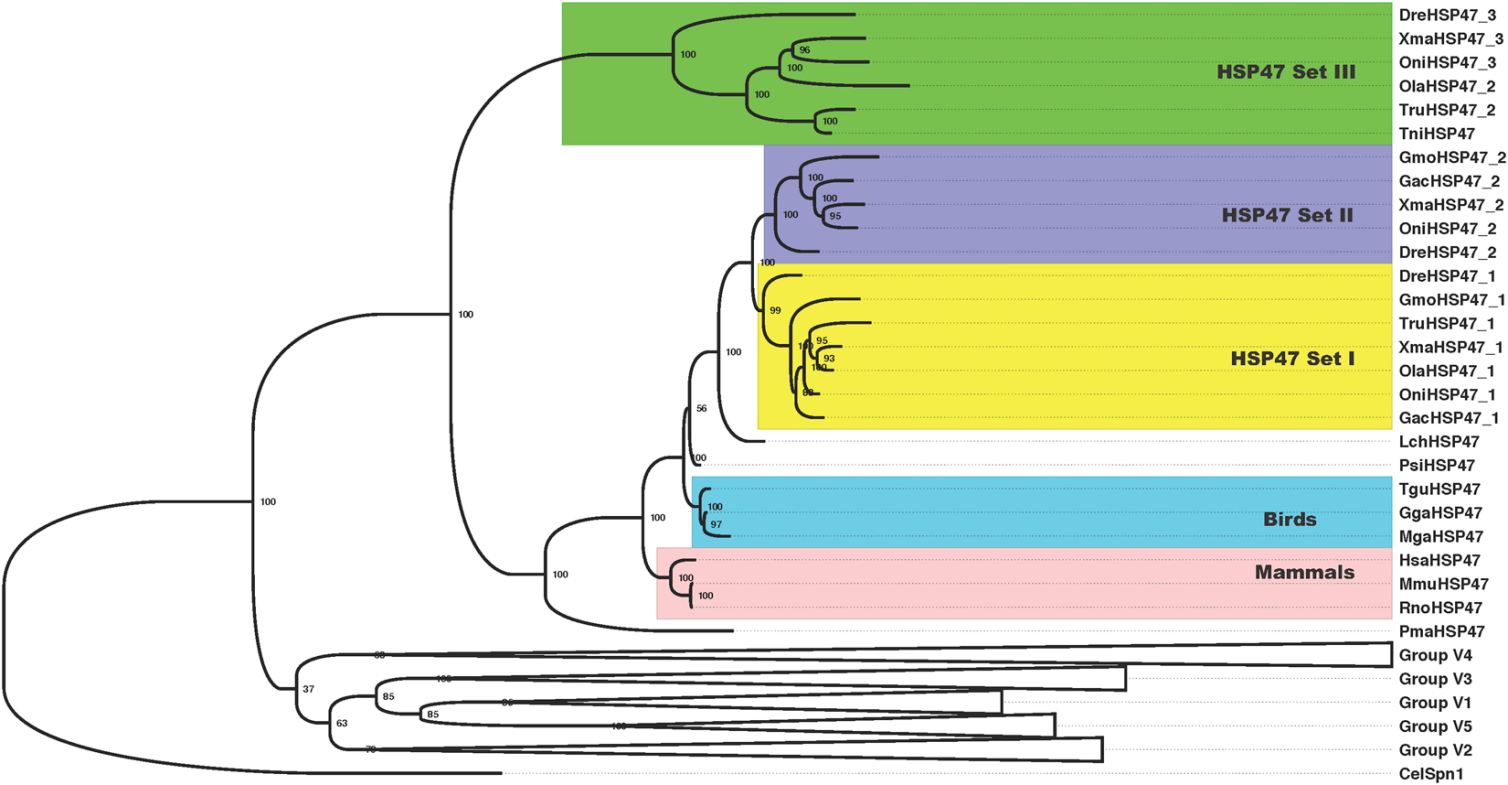
Bayesian phylogeny of representative vertebrate serpins depicts ray-finned fishes specific three sets of HSP47/SERPINH1 within group V6. Set I appears to be close to single copy of HSP47/SERPINH1 in tetrapods, coelacanth and lamprey. Set II is recent duplicate of set I, while set III is very early branching out, hints for its ancestral nature.

We identified HSP47/SERPINH1 genes from several vertebrate genomes using homology detection tools. A majority of tetrapods have single copy of HSP47/SERPINH1 gene. Single copy is also detected in the genomes of coelacanth and lampreys. However fishes have variable numbers of HSP47. Two copies each in the genomes of *Takifugu* and medaka (*Oryzias latipes*), while three copies each in the genomes of Atlantic cod (*Gadus morhua*), cave fish (*Astyanax mexicanus*), platyfish (*Xiphophorus maculatus*), spotted gar (*Lepisosteus oculatus*), and zebrafish (*Danio rerio*) (Table S1). Interestingly, amazon molly (*Poecilia formosa*) has four copies of HSP47/SERPINH1 genes (Table S1). These HSP47/SERPINH1 genes forms three clusters on the Bayesian phylogenetic tree of vertebrate serpins and we have named them as sets I-III (Figs 1 and S1). Set I shares ancestry with single copy of HSP47/SERPINH1 from tetrapods, coelacanth and lamprey. Set II branches out closely with set I, which illustrates that set II is the recent duplicate of set I. Set III is highly diverged in this tree (Figs 1 and S1).

### Variation in the gene structures of HSP47/SERPINH1 genes with HSP47_1 possesses intron insertion in selected ray-finned fishes

Eukaryotic genes are characterized by sets of exons and introns. Intron insertion is creation of new intron in a gene and it can be illustrated as splitting of an exon. Similarly, intron loss is depicted by fusion of two exons into one exon. As rare events, intron insertion and loss are also considered as rare genetic markers^1, 4, 5^.

**Figure 2:**
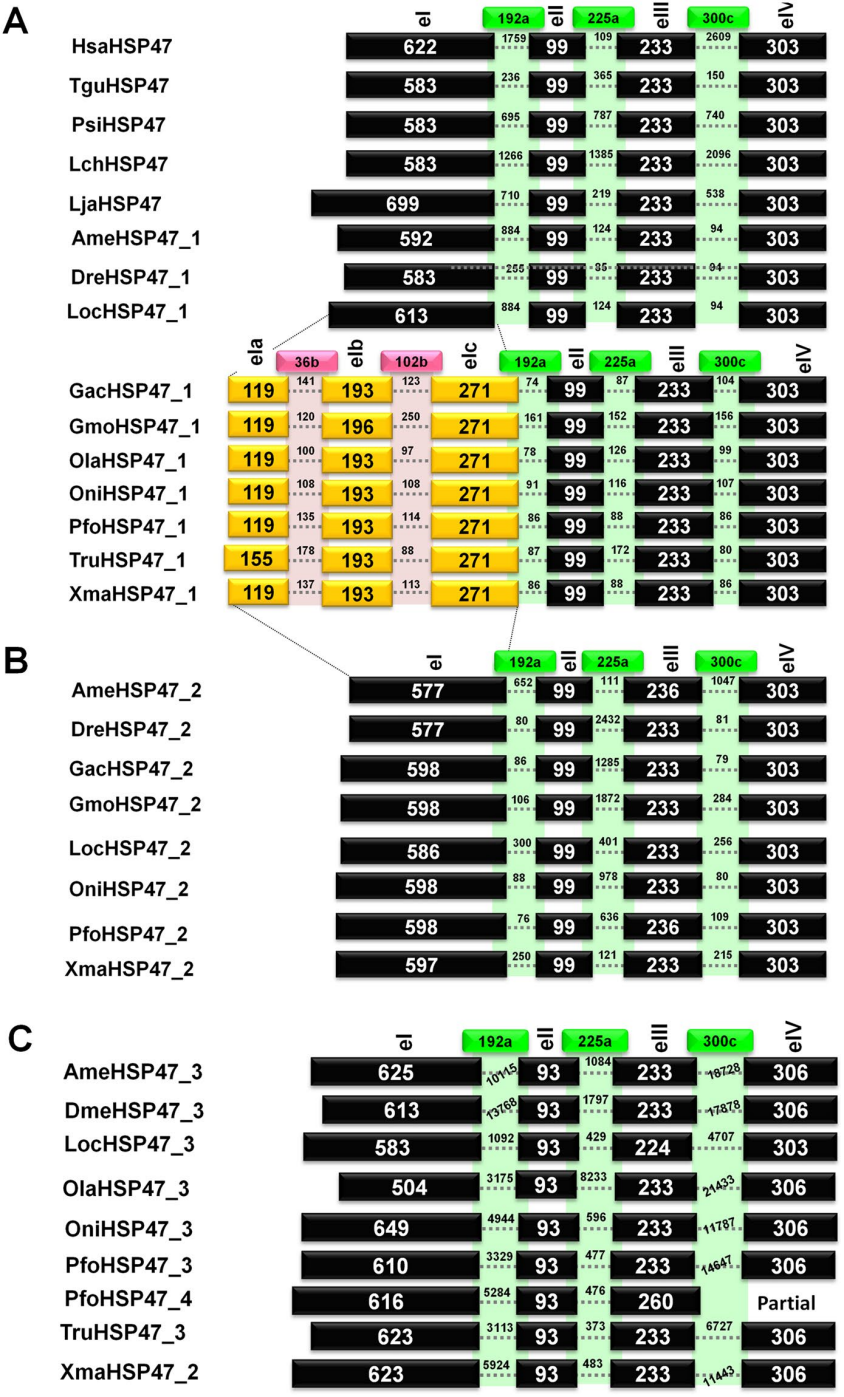
Gene structural patterns of different HSP47/SERPINH1 genes illustrate that intron insertion is only confined to HSP47_1 of selected ray-finned fishes.

Human HSP47/SERPINH1 (HsaHSP47) gene has 4exons/3introns gene structure pattern with conserved introns at positions 192a, 225a, and 300c (numbering according to human α_1_-antitrypsin), which form exons eI-eIV (Fig. 2). Same exon/intron patterns are found in the HSP47/SERPINH1 gene from other tetrapods (such as TguHSP47/SERPINH1 and PsiHSP47/SERPINH1). Similarly, this pattern is also shared by HSP47/SERPINH1 gene from coelacanth, lampreys, cave fish (*A. mexicanus*), spotted gar (*L. oculatus*) and zebrafish (*D. rerio*). The exon sizes are conserved as exons eII-eIV are 99 bp, 233 bp and 303 bp in all analyzed HSP47/SERPINH1 gene in set I and only exception is noted for exon eI with variations ranging from 583 bp (for PsiHSP47/SERPINH1 and LchHSP47/SERPINH1) to 699 bp (for LjaHSP47/SERPINH1).

However, we found changes in the HSP47/SERPINH1 genes from selected ray-finned fishes with two introns inserted in the largest exon eI at the positions 36b and 102b, which formed three small exons as eIa-eIb. These exons have size in the range of 119–155 bp, 193–196 bp and 271 bp, respectively. Intron sizes of two introns at the positions 36b and 102b are in the range of 100 bp (OlaHSP47_1) to 141 bp (GacHSP47) and 88 bp (TruHSP47_1) to 250 bp (GmoHSP47_1) respectively. These two introns are localized in the helices hA and hD upon plotting on protein structural elements (Fig. S2). Remaining three exons (eII-eIV) are of same size as in tetrapods while intron sizes are smaller than their tetrapod counter parts.

Overall, three conserved introns at the positions 192a, 225a and 300c are in ranges of 78 bp (OlaHSP47_1) to 1759 bp (HsaHSP47), 85 bp (DreHSP47_1) to 1385 bp (LchHSP47), and 80 bp (TruHSP47_1) to 2096 bp (LjaHSP47), respectively.

Set II of the HSP47/SERPINH1 gene from fishes possesses 4exon/3introns gene architecture with exon sizes of 577–597 bp, 99 bp, 233–236 bp and 303 bp for exons eI-eIV respectively. Set II HSP47/SERPINH1 have three introns of the length of introns at positions 192a, 225a and 300c as 88–652 bp, 121–2432 bp and 80–1047 bp, respectively.

Set III is characterized by sizes of the exons with variable size of the exon eI, being in the range of 504–649, while exons eII, eIII and eIV have constant size of 93 bp, 233 bp and 306 bp. Intron lengths of the third set of HSP47/SERPINH1 are larger than that of first two sets. The intron at the position 192a has a variable size from 695 bp (in PfoHSP47_4) to 13.768 kb (in DmeHSP47_3). Size of the intron at the position 225a is range from 373 bp (TruHSP47_2) to 8233 bp (OlaHSP47_3). Intron size of the intron at the position 300c is largest amongst all introns of HSP47/SERPINH1 genes analyzed with range of 740 bp (PfoHSP47_4) to 21.433 kb (OlaHSP47_3).

In *Tetraodon* HSP47/SERPINH1 gene (TniHSP47) the intron at position 192a was not identified, probably due to sequencing errors in the coding region of this gene.

Overall the size of exon eI is variable as it can create the 5′ extensions, while size of exon eII (99 bp) is conserved in sets I and II, but varied to 93 bp in the set III HSP47.

The size of exon eIII is conserved in all analyzed HSP47/SERPINH1 genes with exception of AmeHSP47_2 (set II), LocHSP47_3 (set III) and PfoHSP47/SERPINH1 (set III), but PfoHSP47/SERPINH1 is partial. Similarly, size of exon eIV is conserved in the sets I and II (303 bp), but differed by one codon in set III HSP47/SERPINH1 (306 bp). Notably, two introns were inserted in the HSP47/SERPINH1 set I from selected ray-finned fishes.

### Ancestral locus of HSP47/SERPINH1 gene is detected in Japanese lamprey, *Lethenteron japonicum*

Genomic locations and comparisons of syntenic maps provides good source of genetic novelty across organisms. Gene duplications are excellent sources of gene-wide variations and there are two types of gene duplications namely intra-chromosomal and inter-chromosomal; and duplicated genes undergo different fates^6^.

**Figure 3:**
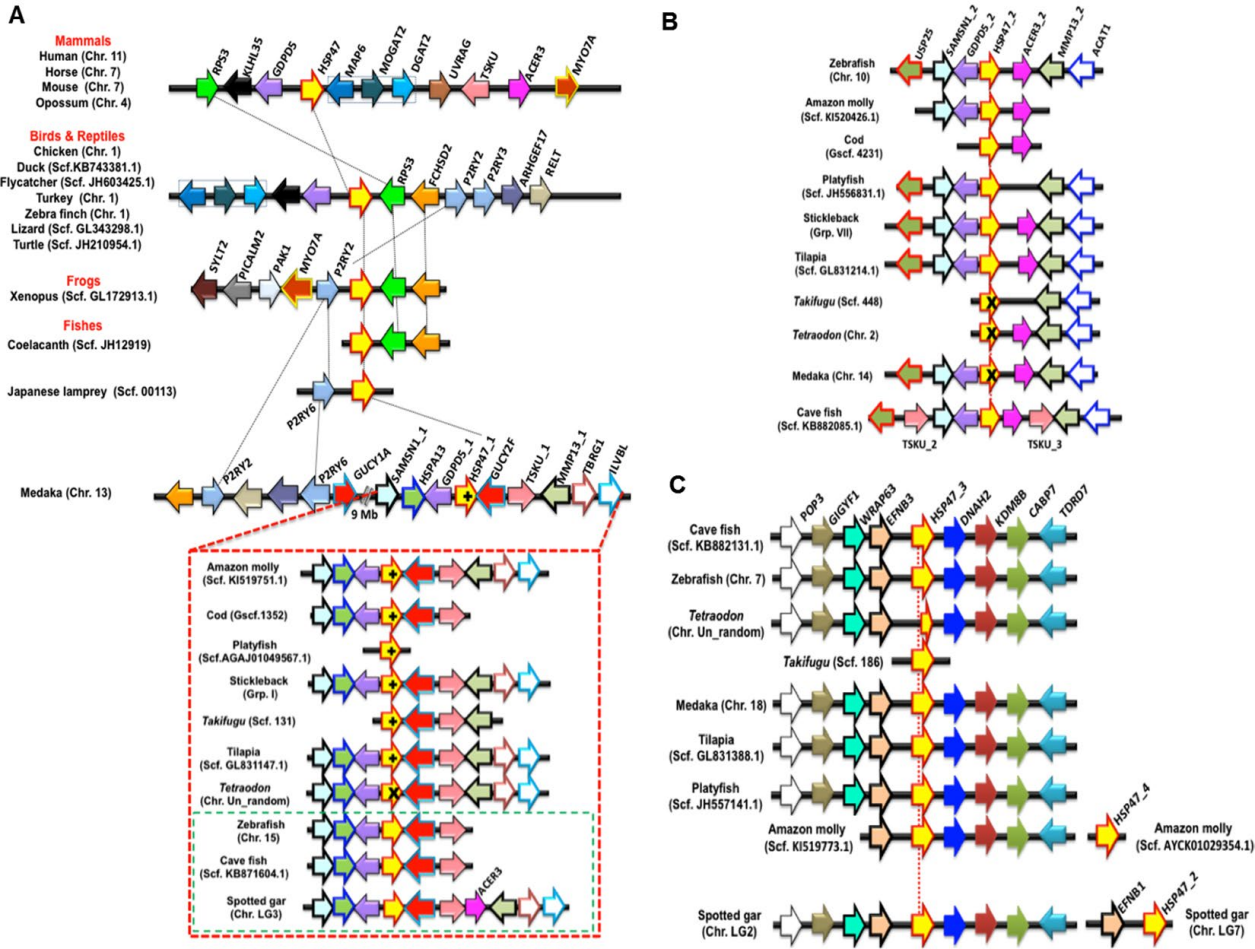
Synteny analyses depict origin of different HSP47/SERPINH1 genes. (**A**) Orthology is shared by tetrapod HSP47/SERPINH1 gene and ray-finned specific HSP47_1/SERPINH1 gene and selected ray-finned fishes have intron gain. Tetrapod HSP47/SERPINH1 shares loci with ray-finned specific HSP47_1. HSP47_1 locus is conserved in different ray-finned fishes as shown in the red box, but not all ray-finned fishes intron gain and fishes with no intron gain are shown in green box. (**B**) HSP47_2 is originated by recent duplication of HSP47_1. (**C**) Locus of HSP47_3 is distinct with only few conserved marker genes. + = presence of two additional introns at the positions 36b and 102b; X = Gene is either partial or lost.

The HSP47/SERPINH1 gene in the human genome is localized on the chromosome 11 flanking by triad of the genes RPS3-KLH35-GDPD5 (Table S2) on the one side, while the other side is occupied by the heptad of genes (MAP6-MAGAT2-DGAT2-UNRAG-TSKU-ACER3-MYO7A (Table S2 and Fig. 3A). This gene clustering is conserved in several mammalian genomes such as in horse (chromosome 7), in mouse (chromosome 7), in opossum (chromosome 4). This genomic organization is also conserved in several birds and known reptile genomes with some variations. In birds and reptiles, the gene RPS3 shifted to other side and similarly, a gene triad (MAP6-MAGAT2-DGAT2, blue gene sets) shuffled its position in the first side. Additional genes are found conserved next to RSP3 gene in the avian and reptile genomes namely, FCHSD2, two P2RY receptors (P2RY2 and P2RY3), ARHGEF17 and RELT genes.

Upon examining frog genome, we found this locus conserved with shuffling of one of the P2RY receptor (P2RY2) to the other side, along with MYO7A (conserved in mammalian cluster) plus some other genes (SYLT2-PICALM2-PAK1), whereas on the second side, two genes are RSP3 and FCHSD2 are conserved.

The genomic assembly of European lamprey (Pmarinus_7.0) is fragmented and hence it is not sufficient to deduce this genomic organization. However, we took advantages of recently sequenced Japanese lamprey (*L. japonicum*) genome. We predicted genes in 1 Mb fragment flanking LjaHSP47/SERPINH1 on the scaffold00131 (KE993802.1:686000–1685660) using Augustus 3.0 ^7^. We have annotated 45 genes (Tables 1 and S3) on this scaffold using BLAST2GO 3.0 ^8^. We are able to deduce a locus with P2RY6 receptor (P2RY6, g19.t1) and LjaHSP47/SERPINH1 (g32.t1). This locus serves ancestral locus of the HSP47. Hence, HSP47/SERPINH1 and its locus are dated back to ~500 million years ago (MYA).

**Table 1:** Summary of gene annotation for the flanking genes on the ancestral locus of HSP47/SERPINH1 on the scaffold00131 from Japanese lamprey (*L. japonicum)* genome, A total 45 genes are residing on this locus of size 1 Mb. The gene g32.t1 is LjaHSP47/SERPINH1 and the g19.t1 is P2RY6-like GPCR (also known as lysophosphatic acid receptor, LPA6R) and these two genes are conserved in several vertebrate genomes (Fig. 3) and hence marked in red color. Gene annotation was performed using BLAST2GO 3.0 ^8^.

Gene ID	Gene Annotation^#^	Protein Length	e-Value	Mean Similarity
**g1.t1**	diacylglycerol kinase partial	99	5,40E − 57	96%
**g2.t1**	—NA—^$^	109	—	—
**g3.t1**	—NA—	108	—	—
**g4.t1**	phosphatidylinositol-glycan biosynthesis class f protein	207	2,10E − 33	76,05%
**g5.t1**	ovotransferrin-like	794	0,00E + 00	58,80%
**g6.t1**	conserved oligomeric golgi complex subunit 3	913	0,00E + 00	74,25%
**g7.t1**	hypothetical chloroplast rf2	813	2,70E − 18	50,30%
**g8.t1**	glypican- partial	202	3,80E − 07	68,55%
**g9.t1**	glypican-5 isoform × 25	537	1,40E − 101	60,35%
**g10.t1**	endoplasmic reticulum-golgi intermediate compartment protein partial	161	1,20E − 86	88,15%
**g11.t1**	endoplasmic reticulum-golgi intermediate compartment protein 3 isoform × 1	266	2,50E − 89	76,95%
**g12.t1**	progestin and adipoq receptor family member 9	354	4,10E − 74	59,30%
**g13.t1**	procollagen c-endopeptidase enhancer 2	389	3,10E − 64	52,75%
**g14.t1**	short transient receptor potential channel 1	497	0,00E + 00	84,20%
**g15.t1**	inhibitor of nuclear factor kappa-b kinase-interacting protein isoform × 1	254	5,30E − 04	47,44%
**g16.t1**	ninein-like protein	366	4,70E − 12	48,70%
**g17.t1**	nucleoredoxin-like protein 2	128	2,00E − 15	55,45%
**g18.t1**	—NA—	169	—	—
**g19.t1**	lysophosphatidic acid receptor 6, P2RY6-like GPCR*	**404**	**2,60E** − **54**	**57,85%**
**g20.t1**	ef-hand calcium-binding domain-containing protein 2	82	5,50E − 26	85,10%
**g21.t1**	—NA—	70	—	—
**g22.t1**	coiled-coil domain-containing protein 160	326	7,20E − 17	47,40%
**g23.t1**	low quality protein: wd repeat-containing protein 78	665	0,00E + 00	59,65%
**g24.t1**	growth hormone secretagogue receptor type 1	260	4,60E − 54	67,65%
**g25.t1**	fibronectin type iii domain-containing protein 3b	843	2,00E − 152	50,75%
**g26.t1**	—NA—	438	—	—
**g27.t1**	fibronectin type iii domain-containing protein 3b	94	4,80E − 08	70,71%
**g28.t1**	—NA—	201	—	—
**g29.t1**	—NA—	423	—	—
**g30.t1**	glycerol kinase 5	532	9,10E − 175	64,60%
**g31.t1**	zinc finger b-box domain-containing protein 1	236	1,70E − 14	42,65%
**g32.t1**	HSP47/SERPINH1*	**470**	**1,60E** − **105**	**68,05%**
**g33.t1**	lysosome-associated membrane glycoprotein 1	380	3,30E − 32	40,05%
**g34.t1**	haus augmin-like complex subunit partial	355	8,10E − 08	63,10%
**g35.t1**	—NA—	225	—	—
**g36.t1**	adp-ribosylation factor	208	3,10E − 53	67,15%
**g37.t1**	rcc1 and btb domain-containing protein 1	531	0,00E + 00	83,15%
**g38.t1**	neuroligin-3 isoform × 4	656	0,00E + 00	66,25%
**g39.t1**	neuroligin- x-linked-like	187	2,50E − 69	70,55%
**g40.t1**	neuroligin-2-like isoform × 5	164	2,50E − 57	84,95%
**g41.t1**	—NA—	96	—	—
**g42.t1**	protein ect2 isoform × 1	368	1,40E − 35	55,80%
**g43.t1**	hypothetical protein H310_04227	72	7,30E − 04	61%
**g44.t1**	protein ect2 isoform × 1	1002	5,30E − 101	73,35%
**g45.t1**	gpalpp motifs-containing protein 1	320	7,90E − 56	55,25%

^#^Full details available in Table S2.

^$^—NA—– Not available.

*Used in Fig. 3, matching to syntenic data.

We started thinking what has happened to this locus in ray-finned fishes and examined several ray-finned fishes. We deduced this locus on the chromosome 13 in medaka (*O. latipes*) genome, 9 Mb away from the current locus of HSP47_1 with two P2RY receptors (P2RY2 and P2RY6), RELT, ARHGEF17 and GUCY1A. This suggests that current locus of HSP47_1 is formed by shuffling of HSP47/SERPINH1 gene along with GDCD5 and GUCY-like gene, which is known as GUCY2F in the new locus. This locus is intact in several fishes with a triad of genes (SAMSN1_1, HSPA13, and GDPD5_1) on the one side, while other side has pentad of genes (GUCY2F, TSKU_1, MMP13_1, TBRG1 and ILBL). This fragment is fully conserved (Fig. 3A) in following fishes namely, amazon molly (scaffold KI519751.1), stickleback (group I), tilapia (scaffold GL831147.1) and spotted gar (chromosome LG3). These flanking genes are not able to deduce in of platyfish (*X. maculatus*) genome (scaffold AGAJ01049567.1), while these partially deduced in the genomes of Atlantic cod, *G. morhua* (gene scaffold 1352), cave fish (scaffold KB71604.1), *Takifugu* (scaffold 131) and zebrafish (chromosome 15). However, selected ray-finned fishes have two extra intron insertions at the positions 36b and 102b, which are marked by + . Notably, this locus is intact in *Tetraodon* genome (unlocalized chromosomal fragment), but HSP47_1 gene is partially present.

Taken together, it is clear that fish-specific HSP47_1 gene shares the locus with tetrapods, coelacanth and lampreys and hence the set I is conserved in all vertebrates.

### Ray-finned fishes possess additional copies of HSP47/SERPINH1

Ray-finned fishes have a duplicated copy of HSP47_1, known as HSP47_2 and it is found that the ray-finned fishes with two triads of genes flanking both sides as seen in chromosome 10 in the zebrafish (*D. rerio*). The first triad of genes is USP25-SAMSM1_2-GDPD5_2 and the second triad comprises ACER3_2, MMP13_2 and ACAT1. This HSP47_2 locus is conserved in several fishes. However, we found that three fishes (*Takifugu, Tetraodon*, and medaka) have lost HSP47_2 within this locus at the scaffold 448, chromosomes 2 and 14, respectively (Fig. 3B).

The set III of HSP47/SERPINH1 is also fish-specific and it is conserved with sets of flanking genes on both side in the cave fish (scaffold KB882131.1), zebrafish (chromosome 7), medaka (chromosome 7), tilapia (scaffold GL831388.1), platyfish (scaffold JH55714.1), amazon molly (scaffold KI519773.1) and spotted gar (chromosome LG2) with tetrad of genes POP3-GIGYG1-WRAP63-EFNB3 flanking on the one side while other side has a tetrad of DNAH2-KDM8B-CABP7-TDRD7. Spotted gar (*L. oculatus*) has HSP47_2 gene on the duplicated locus of HSP47_3 conserved with homolog of ephrin B3 (EFNB3), EFNB1 on the chromosome LG7 (Fig. 3C). Similarly, amazon molly (*P. formosa*) has four copy of HSP47/SERPINH1 gene, known as HSP47_4, which is recently duplicated, but flanking marker genes are not identifiable in the current version of this genome assembly (Version PoeFor_5.1.2).

### Sequence comparisons of group V6 serpins

Protein sequence alignments reflect highly conserved proteins to diverged proteins. Three sets of HSP47/SERPINH1 of ray-finned fishes share three different sequence identity ranges 60–77%, 56–64% and 11–35% with HsaHSP47, respectively (Figs S2 and S3A). The RCL region of all HSP47/SERPINH1 proteins is non-inhibitory (Fig. S3B). However, there are differences in three sets of HSP47, as set III has gaps at the positions P5-P6, while sets I and II HSP47s have as phenylalanine (F)/tyrosine (Y) and isoleucine (I)/valine (V) at the P5 and P6, respectively (Fig. S3B). Additionally, P7-P13 positions where highly variable in the set III, whereas these positions are conserved in the sets I and II HSP47/SERPINH1 with total conservations at the positions P10 [aspartic acid (D)] and P12 [glutamine (N)] and only few mutations at the remaining positions (Fig. S3B). All HSP47/SERPINH1 proteins have an ER retention signal ([RKH]DEL) at the C-terminal ends (Fig. S3C). However, HDEL is only present in set III while RDEL is found majority of set I and II, except for GacHSP47_2, where it is KDEL in the set II. A total of 63 positions are highly conserved with identities between 90–100%, while 83 positions have identities of 70–89%, while 103 positions are identities score from 50–69 (Fig. S2 and Table 2). Out of 51 amino acid positions conserved in the majority of serpins, 39 residues are fully conserved, while three are partly conserved (Fig. S2 and Table 2). There are two N-glycosylation sites at the position 94 and 120 (HsaHSP47/SERPINH1 numbering, Fig. S2) with second being highly conserved at the end of helix hD. Lampreys have extension of 9 residues in the helix D, before the N-glycosylation site (Fig. S2).

**Table 2:** Summary of sequence conservation on the secondary structural element levels of HSP47/SERPINH1 proteins.

**Structural Components**	**Sequence Conservation**			**Status of 51 conserved amino acids** ^**$**^				
	**90–100**	**70–89**	**50–69**					
N-terminal segment	0	8	9					
hA	3	4	7	**F33** ^**!**^				
s6B	1	0	2	N49	S53			
hB	3	4	4	P54	S56	L61	G67	
hC	2	3	2	T72	L80			
hD	2	5	5					
s2A	0	6	3					
hE	1	5	4	F130				
s1A	0	1	2					
hF	3	4	9	F147	I157	N158	**V161**	T165
Loop between hF/s3A	1	1	2	I169	T180			
s3A	5	4	8	L184 ^%^	N186	F190	K191	**G192**
hF1	0	2	1					
s4C	2	0	3	F198	T203	F208		
s3C	5	4	2	V218	M220	M221		
s1B	1	0	1					
s2B	1	2	1	**Y244**				
s3B	0	1	1	L254	P255			
hG	2	4	1					
hH	1	1	2					
s2C	3	2	1					
s6A	0	1	2	P289	K290			
hI	4	0	2	L299	L303	G307		
hI1	0	2	0					
Loop between hI/s5A	6	6	6	**F312**	A316	L327		
s5A	2	3	1	H334	**E342**			
s4A (RCL)	3	4	10	G344	**A347**			
s1C	3	0	0					
s4B	2	3	2	P369	F370			
s5B	2	2	5	L383	F384	G386		
C terminal end	4	1	5	P391				

^$^As proposed by Irving *et al*. (2000). ^!^Bold – Missing.

^%^Underscore – Partially present (<50% of HSP47).

## Discussion

**Figure 4:**
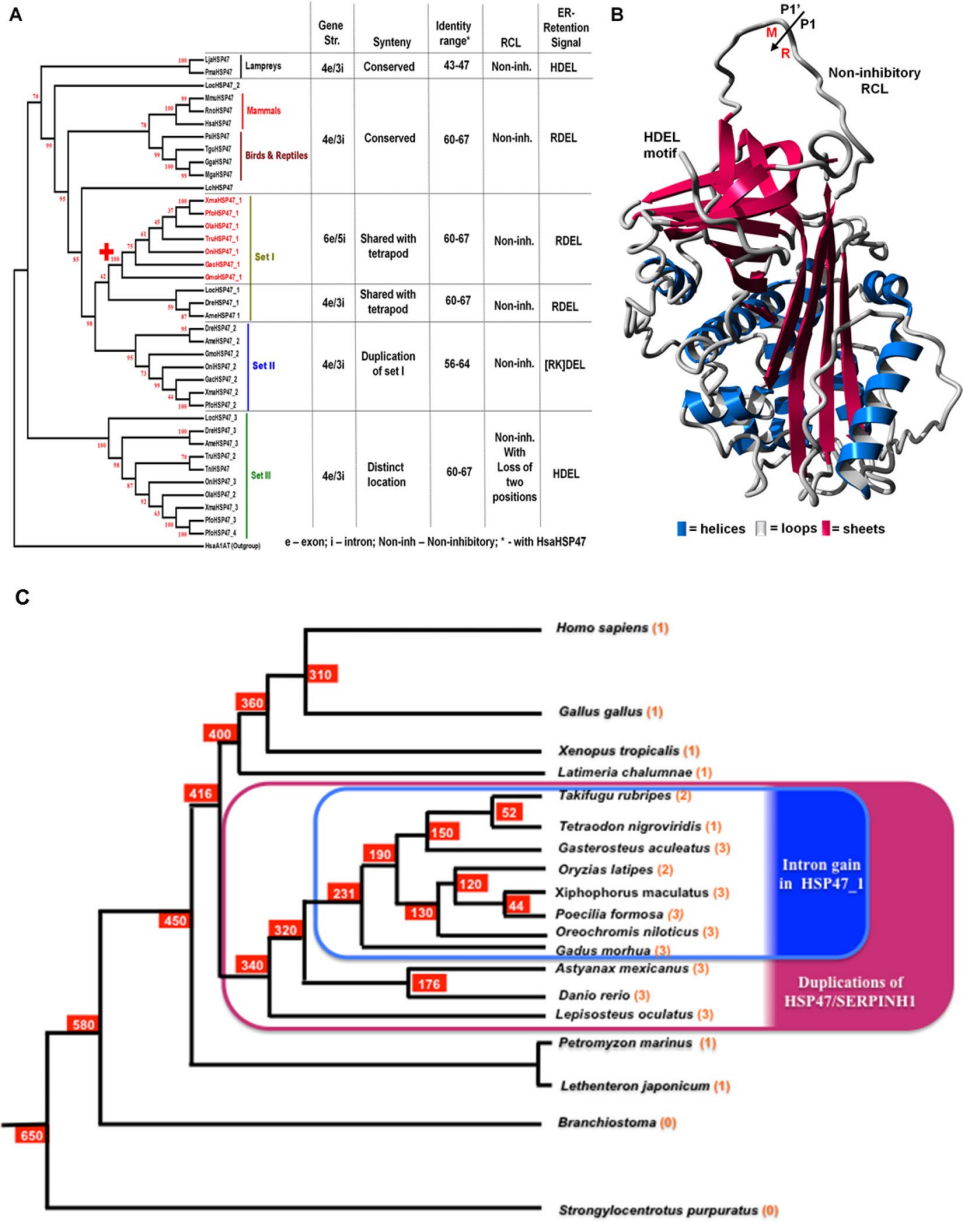
Collagen-specific molecular chaperone HSP47/SERPINH1 has originated in lampreys, dating ~500 million years ago (MYA) and ray-finned fishes has 1–4 copies of this chaperone. (**A**) Characteristics of different HSP47/SERPINH1 as depicted with the Neighbor-Joining tree. Except for HSP47/SERPINH1 set I from selected ray-finned fishes (marked red). All HSP47/SERPINH1 genes have 4exon-3intron gene structures with variable syntenic organization and sequence identities. All HSP47/SERPINH1 proteins possess non-inhibitory RCL and ER-retention signals with some variations. (**B**) Homology model of ancestral HSP47/SERPINH1 from Japanese lamprey, illustrating non-inhibitory RCL and ER-retention signal as the HDEL motif. (**C**) Timescales for the Evolutionary history of HSP47/SERPINH1 depicts origin of HSP47 duplications (pink shade), dated between 416–360 MYA and intron insertion events for HSP47_1 (blue shade), dated between 231–190 MYA. Number of HSP47 is shown in bracket.

HSP47/SERPINH1 is a critical regulator of the collagen maturation and associated embryonic development. However, despite great efforts on discovering the molecular mechanisms and clinical relevance of HSP47/SERPINH1 gene and protein functions, there is no independent comprehensive study on molecular phylogeny of HSP47/SERPINH1. The current study provides an updated repository of the HSP47/SERPINH1 gene from 61 vertebrate species (Table S1) and summarizes major concepts revolving around sequence, structure and phylogeny of HSP47/SERPINH1 across vertebrate genomes. We have summarized overall finding of this work in Fig. 4. Tetrapods have single copy of collagen-specific HSP47/SERPINH1 (Table S1 and Fig. 4A); also in coelacanth and lampreys (Fig. 4). However, this is not the case in ray-finned fishes, which possesses three sets of HSP47/SERPINH1 genes exception amazon molly (*P. formosa*) has four copies while *Tetraodon* has only one partial copy (Table S1 and Fig. 4A). We identified the ancestral genomic loci of HSP47 in the Japanese lamprey (*L. japonicum)* genome (Fig. 3 **)**. Ancestral HSP47/SERPINH1 protein from Japanese lamprey (*L. japonicum)* encodes for 470 amino acid long and it is named as LjaHSP47/SERPINH1, which has non-inhibitory RCL and HDEL motif at the C-terminal end (Fig. 4B). The LjaHSP47/SERPINH1 has 47% sequence identity with HsaHSP47/SERPINH1 (Fig. S3). Along with locus and full-length protein, it is assured that it is functionally active gene in the lamprey genome.

Set I and II are very close to each other in terms of sequence identities and syntenic similarities (Figs S3 and 4A), in contrast to set III. This indicates recent duplications in ray-finned fishes. Upon scaling evolutionary time scale of HSP47 duplications and intron insertions, it become clear that ray-finned fishes specific HSP47 duplication events have occurred between 416–360 MY. In contrast, HSP47–1 specific intron insertions can be dated back to 231–190 MY (Fig. 4C).

HSP47/SERPINH1 gene is characterized by four exons eI-eIV in majority of vertebrates in the core serpin domain, while selected ray-finned fishes (red color near + sign in Fig. 4A) have split into the exon eI by two intron invasions at the positions 36c and 102b forming smaller exons eIa-eIc (Fig. 2A). This gives rise to changes into exon/intron pattern from 4e/3i to 6e/5i (Fig. 4A). This pattern change is limited only to first copy of HSP47/SERPINH1 (HSP47_1), but not to second and third copies in selected fishes. Eukaryotic genes are expressed as pre-mRNAs that are converted to mRNA by splicing mechanisms, which removes introns and exons, creating expressing segment of the genes^9^. Spliceosomal introns and its splicing machinery are hallmarks of eukaryotic genomes. However, the mystery about their creation remains puzzling^10^. There are total 24 conserved introns in vertebrate serpins encompassing group V1-V6^4^ with six additional introns that were gained in selected ray finned fishes among serpin genes^11^. Trademarks of genome evolution are several types of gene rearrangements, such as inversions, translocations, duplications and transpositions. Gains of introns are normally coupled by these events. There are seven different mechanisms have been proposed for intron gain/invasions^12, 13^. Genome compaction and associated double-strand break repair (DSBR) were accountable with several examples of intron creations in selected ray-finned fishes whose genome underwent compaction events in the serpin superfamily^11^ and in the GPCR superfamily^5^. These repair processes involved in successful genome compaction best-explained gains of introns in ray-finned fishes.

The HSP47/SERPINH1 gene is conserved on the same locus from lamprey to human along with P2RY6-like GPCR for about ~500 MY (Fig. 3). Ray-finned fishes have three copies of HSP47/SERPINH1 and these are originated via chromosomal duplications (Fig. 3). Additionally, fishes have differential presences of HSP47, such as Amazon molly (*P. formosa*) has 4 copies, while *Tetraodon* genome has single copy of HSP47, named as TniHSP47/SERPINH1 (Table S1). However, this gene is remained partial in different version of genome assemblies of *Tetraodon* ^14^. However, lack of full HSP47/SERPINH1 in *Tetraodon* appears to be a problem of genomic assembly as closely related *Takifugu* has two copies of HSP47/SERPINH1 (Table S1, Figs 3 and S2). This is also supported by the fact that vertebrates needs HSP47/SERPINH1 for collagen assemblies and recently it is shown that HSP47_1 is essential for skeletal growth and patterning during fin regeneration in zebrafish (*D. rerio*)^15^. Hence, missing HSP47/SERPINH1 genes will pose severe implications of morphology of fishes.

HSP47/SERPINH1 is the group V6 member^1^, which has variations in numbers by chromosomal duplications in ray-finned fishes (Fig. 3). However, these genes remained in single copy of each locus (Fig. 3). There are other examples of serpins, which remained single gene in the chromosomal fragments (for examples, angiotensinogen^16^, heparin cofactor II^17^, and antithrombin III^18^, whereas large-scale tandem duplications on the same loci lead into several paralogs for groups V1 (serpinBx^19^) and V2 (serpinAx^20^).

HSP47 is highly specific non-inhibitory serpin, which serve as collagen-specific chaperone originating in lamprey to human. This gene is missing in any invertebrate, whose genomes are known like urochordate serpins^17^, and or cephalochordate serpins^11, 14^. The genome of sea urchin (*Strongylocentrotus purpuratus*) harbors 10 inhibitory serpins named as Spu-spn-1 to Spu-spn-10 ^14^. Similarly nematode model, *Caenorhabditis elegans* has 8 inhibitory serpins^21^. It holds true with insect serpins like in house fly (*Drosophila melangoster*)^22^ and Colorado potato beetle (*Leptinotarsa decemlineata*)^23, 24^.

In our systematic comparative genomic surveys of all serpins^14^, we found that only a single vertebrate serpin called neuroserpin can traced back in the invertebrate genomes, and it is only in sea urchin and cephalochordate based on genomic mapping and sequence based characters^4, 14^.

Hence by and large, invertebrate serpins are distinctive in many features (including genomic locations, gene structures and sequence identities) from any vertebrates. This corroborates that collagen-specific chaperone HSP47 is only limited to vertebrates. There are 3–4 copies of HSP47 in fishes, but roles of different copies of HSP47 are not known in any actinopterygian model. Therefore, it is difficult to pinpoint what are the biological significances of these duplicated HSP47. However based on the available literature, we can corroborate that ray-finned fishes have duplications of collagen genes - col1a1, col2a1, col5a2, col5a3, col11a1 and col27a1 and mostly likely duplicated HSP47 genes are required for proper folding of these duplicated collagen genes^25^. However to confirm this point, a detailed investigation of co-evolution of collagens and their chaperones is required. Origins and features of fin skeleton of the ray-finned fishes have been controversial^26^. This is because extracellular matrix of fin skeleton depicts hybrid characteristics of both bone and cartilage. This complexity is enhanced by the presence of several duplicated collagen genes^25^ and 3–4 paralogs of their chaperones. This requires several lines of investigation of fish fin morphogenesis and developments with roles of different collagen and their chaperones.

Human HSP47/SERPINH1 is associated several human diseases like rheumatoid arthritis, where autoantibodies to HSP47 protein have been found in rheumatoid arthritis patients^27^. Similarly a genetic variant of HSP47/SERPINH1 is associated with a severe and lethal form of osteogenesis imperfecta (OI)^28^. Recently, it is shown that increased Hsp47 expression promotes breast cancer progression by enhancing deposition of ECM proteins^29^. Our current study is beyond scope of directly resolving disease roles of HSP47/SERPINH1. However, it hints for possibilities that investigated properly zebrafish (*D. rerio*) can serve as a model to study HSP47/SERPINH1 based diseases in future.

In summary, this study provides updated repository of HSP47/SERPINH1 genes and summarizes some concepts revolving around sequence, structure and phylogeny of group V6 serpins. We have identified three sets of HSP47/SERPINH1 gene in ray-finned fishes and ancestral locus in the Japanese lamprey genome.

## Methods

### Collection of HSP47/SERPINH1 using BLAST suite

We collected genomic DNA and protein sequences from different vertebrate genomes via Ensembl release 76 (August 2014)^30^ using BLAST suite (E-value < 1e-10) for HSP47/SERPINH1 (Table S1).

### Gene prediction and intron characterization of HSP47/SERPINH1

To ensure accuracy of HSP47/SERPINH1 gene structure, we combined gene structure predictions from Ensembl^30^ with that of AUGUSTUS 3.0 suite^7^. Mature human α_1_-antitrypsin was used as standard sequence for intron position mapping and numbering of intron positions, followed by suffixes a–c for their location as reported previously^1, 4, 5^.

### Construction of Synteny maps for different HSP47/SERPINH1 genes

We carried out multi-species synteny analyses for HSP47/SERPINH1 genes using Ensembl genome browser^30^ and the Mapviewer from the NCBI (Website https://www.ncbi.nlm.nih.gov/mapview/).

### Detection of ancestral HSP47/SERPINH1 locus and characterization of the flanking genes

After homology screening of HSP47/SERPINH1 gene in the Japanese lamprey *(L. japonicum*) genome we downloaded 1 Mb region flanking LjaHSP47/SERPINH1 on the scaffold00131 from webpage of the Japanese lamprey genome project (http://jlampreygenome.imcb.a-star.edu.sg/). We have predicted genes on this 1 Mb region with using AUGUSTUS 3.0 suite^7^ with training dataset of *Petromyzon marinus*, which yielded 45 genes. Furthermore, we performed gene annotation for these 45 genes using BLAST2GO 3.0^8^.

### Sequence analyses of HSP47/SERPINH1 proteins

We constructed HSP47/SERPINH1 protein alignment using the MUSCLE^31^ and visualized with GENEDOC^32^ as shown in Fig. S2. We generated sequence logos of conserved regions of HSP47/SERPINH1 proteins were constructed by Weblogo 3.3^33^.

### Phylogenetic analyses of HSP47/SERPINH1

We built phylogenetic tree of selected serpins by the Bayesian (2 runs, until average standard deviation of split frequencies was lower than 0.0098, 25% burn-in-period) using MrBayes 3.2.1 ^34^ with best fit protein substitution model, WAG [5 categories (+*G*, parameter = 4.61)] as computed in MEGA 5^35^. Additionally, we constructed a Neighbor-Joining tree from selected HSP47/SERPINH1 proteins using MEGA 5^35^.

### Protein modeling of LjaHSP47/SERPINH1 from Japanese lamprey (*L. japonicum)*

We created structural model of LjaHSP47/SERPINH1 from Japanese lamprey using the I-TASSER^36^ and we visualized the resulting model using YASARA^37^.

## Electronic supplementary material

Supplementary Information
