# Supplementary material for: Ancestry & molecular evolutionary analyses of heat shock protein 47 kDa (HSP47/SERPINH1)
Source: Sci Rep. 2017 Sep 4;7:10394. doi: 10.1038/s41598-017-10740-0 (PMC5583329; doi:10.1038/s41598-017-10740-0)
Supplement: Supplementary file 1 — Supplementary Information [file 41598_2017_10740_MOESM1_ESM.pdf]

Ancestry & molecular evolutionary analyses of heat shock protein 47 kDa (HSP47/SERPINH1)

Abhishek Kumar[1,2*] Anita Bhandari[3], Sandeep J. Sarde[1,4] & Chandan Goswami[5]

[1]Department of Genetics & Molecular Biology in Botany, Institute of Botany, Christian-Albrechts-University at Kiel, Germany

[2]Current Address - Division of Molecular Genetic Epidemiology German Cancer Research Center, Heidelberg, Germany

[3]Institute for Cardiogenetics, University of Lübeck, Lübeck, Germany

[4]Laboratory of Entomology, Department of Plant Sciences, Wageningen University & Research, Wageningen, Netherland

[5]National Institute of Science Education and Research, Bhubaneswar, Orissa, India

[*]Email:
AK –abhishek.abhishekkumar@gmail.com

Fig S1. Detailed phylogenetic tree of HSP47 proteins reveals three sets of HSP47 in ray-finned fishes. This tree was obtained using MEGA7 with maximum likelihood method using bootstrap of 1000. Ame - Ailuropoda melanoleuca;Apl - Anas platyrhynchos;Aca - Anolis carolinensis;Ame - Astyanax mexicanus;Bta - Bos taurus;Cja - Callithrix jacchus;Cfa - Canis lupus familiaris;Cpo - Cavia porcellus;Csa - Chlorocebus sabaeus;Dre - Danio rerio;Dno - Dasypus novemcinctus;Dor - Dipodomys ordii;Ete - Echinops telfairi;Eca - Equus caballus;Eeu - Erinaceus europaeus;Fca - Felis catus;Fal - Ficedula albicollis;Gmo - Gadus morhua;Gga - Gallus gallus;Gac - Gasterosteus aculeatus;Ggo - Gorilla gorilla;Hsa - Homo sapiens;Itr - Ictidomys tridecemlineatus;Lch - Latimeria chalumnae;Loc - Lepisosteus oculatus;Lja - Lethenteron japonicum;Laf - Loxodonta africana;Mmu - Macaca mulatta;Meu - Macropus eugenii;Mga - Meleagris gallopavo;Mmur - Microcebus murinus;Mdo - Monodelphis domestica;Mmu - Mus musculus;Mfu - Mustela putorius furo;Mlu - Myotis lucifugus;Nle - Nomascus leucogenys;Opr - Ochotona princeps;Oni - Oreochromis niloticus;Ocu - Oryctolagus cuniculus;Ola - Oryzias latipes;Oga - Otolemur garnettii;Oar - Ovis aries;Ptr - Pan troglodytes;Pan - Papio anubis;Psi - Pelodiscus sinensis;Pma - Petromyzon Marinus;Pfo - Poecilia formosa;Pab - Pongo abelii;Pca - Procavia capensis;Pva - Pteropus vampyrus;Rno - Rattus norvegicus;Sha - Sarcophilus harrisii;Ssc - Sus scrofa;Tgu - Taeniopygia guttata;Tru - Takifugu rubripes;Tsy - Tarsius syrichta;Tni - Tetraodon nigroviridis;Tbe - Tupaia belangeri;Ttr - Tursiops truncatus;Xtr - Xenopus tropicalis;Xma - Xiphophorus maculatus;

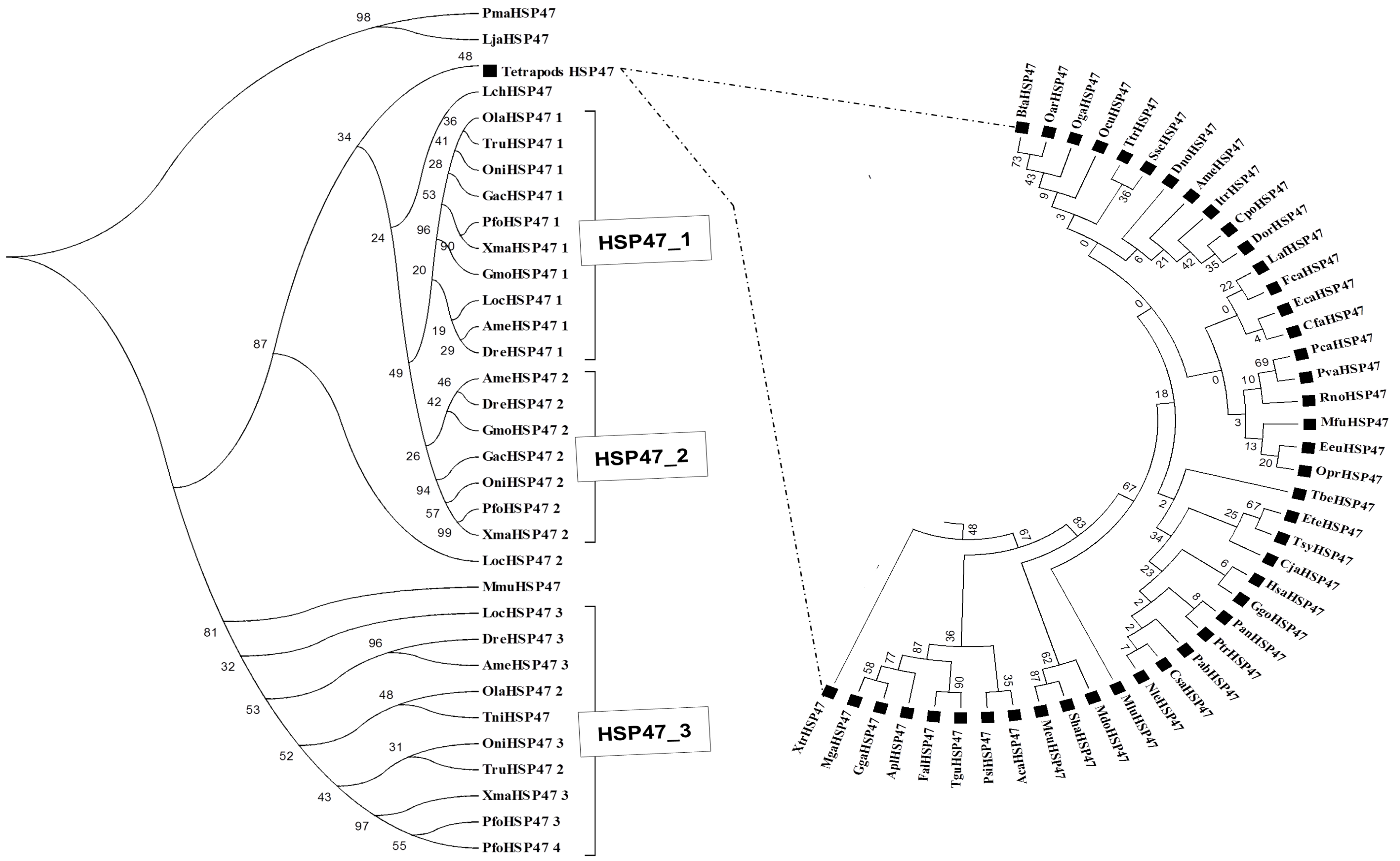
PmaHSP47
LjaHSP47
Tetrapods HSP47
LchHSP47
OlaHSP47 1
TruHSP47 1
OniHSP47 1
GacHSP47 1
PfoHSP47 1
XmaHSP47 1
GmoHSP47 1
LocHSP47 1
AmeHSP47 1
DreHSP47 1
AmeHSP47 2
DreHSP47 2
GmoHSP47 2
GacHSP47 2
OniHSP47 2
PfoHSP47 2
XmaHSP47 2
LocHSP47 2
MmuHSP47
LocHSP47 3
DreHSP47 3
AmeHSP47 3
OlaHSP47 2
TniHSP47
OniHSP47 3
TruHSP47 2
XmaHSP47 3
PfoHSP47 3
PfoHSP47 4
HSP47_1
HSP47_2
HSP47_3
BtaHSP47
OarHSP47
OgaHSP47
OcuHSP47
TtrHSP47
SscHSP47
DnoHSP47
AmeHSP47
CpoHSP47
DorHSP47
LafHSP47
FcaHSP47
EcaHSP47
CfaHSP47
PvaHSP47
RnoHSP47
MfuHSP47
EeuHSP47
OprHSP47
TbeHSP47
EteHSP47
TsyHSP47
CjaHSP47
HsaHSP47
GgoHSP47
PanHSP47
PtrHSP47
PabHSP47
CsaHSP47
NleHSP47
MluHSP47
MdoHSP47
ShaHSP47
MeuHSP47
AcaHSP47
TguHSP47
FalHSP47
GgaHSP47
MgaHSP47
XtrHSP47

Fig S2. Sequence alignment of different HSP47 proteins. Percentage identity scores of 90-100, 70-89, 50-69 are marked by black, grey and yellow shades, respectively. Known conserved (≥70%) amino acids (proposed by Irving et al 2000) are marked by different colors of hashes (above the alignment), #, # and # for presence, absence and partial presence (<50% of HSP47 proteins) of residues, respectively. Structural elements are marked above the alignment: **α**-helices and **β**-sheets as ~~ and ==>, respectively. Three serpin motifs are shown with black boxes. Intron position and phasing are shown above alignment with green and cyan shades for standard introns and additional intron insertions (confined to only HSP47_1 of selected ray-finned fishes at the positions 36b and 102b), respectively. @@@ indicates N-glycosylation site (NxT, where x - any residue).

```
HsaHSP47    1  ------------------------------MRSL---------LLLSAFC   11
MmuHSP47    1  -------------------------------MRS---------LLLGTLC   10
RnoHSP47    1  -------------------------------MRS---------LLLGTLC   10
GgaHSP47    1  -------------------------------MQI---------FLVLALC   10
TguHSP47    1  -------------------------------MWI---------IMGLALL   10
MgaHSP47    1  ----------------------------EGPMWIWDHCT----ASIALMQ   18
PsiHSP47    1  -------------------------------MWV---------TSLLALC   10
LchHSP47    1  -------------------------------MWM---------TRLLSLS   10
AmeHSP47_1  1  -------------------------------MWV---------SKIAALC   10
LocHSP47_1  1  ---------------------IAQLPSITESMWV---------TKLVALS   20
DreHSP47_1  1  -------------------------------MWV---------SSLIALC   10
GacHSP47_1  1  -------------------------------MWM---------TNLVAFC   10
GmoHSP47_1  1  -------------------------------MWM---------TSVLALC   10
OlaHSP47_1  1  -------------------------------MWT---------TTIVALC   10
OniHSP47_1  1  -------------------------------MWM---------TNIVGLC   10
PfoHSP47_1  1  -------------------------------MWM---------TRAVALC   10
TruHSP47_1  1  ----------------------------LRLLWLLWKTETMRAANIVVLS   22
XmaHSP47_1  1  -------------------------------MWM---------TTAVALC   10
DreHSP47_2  1  -------------------------------MLA---------SNVLLLC   10
OniHSP47_2  1  -------------------------------MWV---------TNLVSLV   10
GmoHSP47_2  1  -------------------------------MLV---------TSLVALA   10
XmaHSP47_2  1  -------------------------------MWL---------TLLVSLA   10
GacHSP47_2  1  -------------------------------MWV---------PNLAALV   10
PfoHSP47_2  1  -------------------------------MWL---------TFLVSLA   10
AmeHSP47_2  1  -------------------------------MWV---------KSLLALC   10
LocHSP47_2  1  -------------------------------AMA---------FKAAVVS   10
AmeHSP47_3  1  -------------------------------MLS---------AAVLYLS   10
DreHSP47_3  1  ------------------------------MQPI---------FPVPLLF   11
OniHSP47_3  1  -------------------------------M-----------LPRLPFC    8
OlaHSP47_2  -  --------------------------------------------------    -
TruHSP47_2  1  ----------------------------SRAAML---------PRLPVYI   13
TniHSP47    -  --------------------------------------------------    -
XmaHSP47_3  1  -------------------------------M-----------LFRLAVC    8
LocHSP47_3  1  -------------------------------AML----------LLPATV    9
PfoHSP47_3  1  ----------------------------------------------LAVC    4
PfoHSP47_4  1  ------------------------------RPAM---------LLRLAVC   11
PmaHSP47    1  ------------------------------MLLL---------EALASGA   11
LjaHSP47    1  MGLGFNPTEVRVQRDQDHRHPTELDEQESDGHYA---------THAAAGA   41
             ------------------------------------------N-terminal
```

```
HsaHSP47     12   LLE---AALAAEVK-------KPAAAAAPGT---AEKLSPKAATLAERSA   48
MmuHSP47     11   LLA---VALAAEVK-------KPLEAAAPGT---AEKLSSKATTLAERST   47
RnoHSP47     11   LLA---VALAAEVK-------KPVEATAPGT---AEKLSSKATTLAERST   47
GgaHSP47     11   GLA---AAV-------------------PSE---DRKLSDKATTLADRST   35
TguHSP47     11   GLA---AAV-------------------PSE---DRKLSDKATTLAERST   35
MgaHSP47     19   MLPEPSLAAAV-----------------PSE---DRKLSDKATTLADRST   48
PsiHSP47     11   ALV---AAV-------------------PSE---DKKLSDKAATLADRSA   35
LchHSP47     11   ILA---AVA-------------------ICE---EKKLSEHATTLADRSS   35
AmeHSP47_1   11   LLA---VAS-------------------CAEVKDAKKLSTHATSLADTSA   38
LocHSP47_1   21   LLA---ALA-------------------TAE---EKKLSSHASMLADTSA   45
DreHSP47_1   11   LLA---VAV-------------------SGE---DKKLSTHATSMADTSA   35
GacHSP47_1   11   LLA---VVA-------------------SAE---EKKLSSYATALADHSA   35
GmoHSP47_1   11   LLA---PLA-------------------SAD---GRKLSSHATLLADNSA   35
OlaHSP47_1   11   LLA---LGA-------------------SAE---DKKLSVHATTLADNSA   35
OniHSP47_1   11   LLA---LVA-------------------SAE---DVKLSTHATTLADKSA   35
PfoHSP47_1   11   LLA---FVA-------------------SAE---DKKLSSHANTLADNSA   35
TruHSP47_1   23   LLA---LLA-------------------SAE---DKKLSNHATTLADNSA   47
XmaHSP47_1   11   LLA---FVA-------------------SAE---DKKLSSHANTLADNSA   35
DreHSP47_2   11   LLA---TVSA------------------------NKTLSSIATTLADNSA   33
OniHSP47_2   11   LLT---TAASA--------------ATSTSK---NKVLSNHATILADSTA   40
GmoHSP47_2   11   LLA---HTASS--------------ATSASP---DKVLSTHASLLADNSA   40
XmaHSP47_2   11   LLA---PAISA--------------ATATTA---DKVLSNHATILADNSA   40
GacHSP47_2   11   LLA---TTAAA--------------ATSASP---DQVLSKHATTLADTSA   40
PfoHSP47_2   11   LLA---PAISA--------------ATATTA---DKVLSNHATILADNSA   40
AmeHSP47_2   11   LLA---SVRA------------------------DKALSSHATILVDSST   33
LocHSP47_2   11   FLI---GIV----------------VAE------PVALKAPTSVLSESTV   35
AmeHSP47_3   11   LLNLLIVGV------------GSSSSKAPPATGEPPQPQQASSSFGHPSW   48
DreHSP47_3   12   LLLAQQSVWSS-----------------TPQEPKVQGSPPEISSLHHPTW   44
OniHSP47_3    9   LLISLPVLLVQGSATN-----SSKKSADTPAGPVSRPPPLPPPPQGDPSW   53
OlaHSP47_2    1   --------------------------------------------LSDPSW    6
TruHSP47_2   14   LLF---LPLAP----------VQRSTADSSEKSSASSPHLPPPPLGDPSW   50
TniHSP47      -   --------------------------------------------------    -
XmaHSP47_3    9   LPVSLLVLL------------VQSSTTKSSKSPAASVSPSLRPLVNDPSW   46
LocHSP47_3   10   LWG---VLL-------------------SAH---CKPPGDHAATLAEGSW   34
PfoHSP47_3    5   LLVSLRVLL------------VQSGAATSSQSPAASVSPSLPPLVNDPSW   42
PfoHSP47_4   12   LLVSLRVLL------------VQSGAATSSQSPAASVSPSLPPLVNDPSW   49
PmaHSP47     12   LAA---AAAADGKKATVSKADAAAANNATAP---PKNLSEHAKKVGEGNW   55
LjaHSP47     42   ALASGAAAAAADGKKKATEPKADAAAVTNATAA-PKNLSEHAKKVGEGNS   90
                  segment-------------------------------------~~~~~~
```

```
                    # 36b             #   ##      #      #    #
                       |
HsaHSP47     49    GLAFSLYQAMAKDQAV--ENILVSPVVVASSLGLVSLGGKATTASQAKAV    96
MmuHSP47     48    GLAFSLYQAMAKDQAV--ENILLSPLVVASSLGLVSLGGKATTASQAKAV    95
RnoHSP47     48    GLAFSLYQAMAKDQAV--ENILLSPLVVASSLGLVSLGGKATTASQAKAV    95
GgaHSP47     36    TLAFNLYHAMAKDKNM--ENILLSPVVVASSLGLVSLGGKATTASQAKAV    83
TguHSP47     36    TLAFNLYHAMAKDKDM--ENILLSPVVVASSLGLVSLGGKATTASQAKAV    83
MgaHSP47     49    TLAFNLYHAMAKDKNM--ENILLSPVVVASSLGLVSLGGKATTASQAKAV    96
PsiHSP47     36    TLAFNLYHTMAKDKNT--ENILVSPVVVASSLGLVSLGGKATTASQAKAV    83
LchHSP47     36    TLAFNLYHTMAKEKNM--ENILISPVVVASSLGLVSLAGKASTASQAKAV    83
AmeHSP47_1   39    NLAFNLYHNLAKETSQ--DNILVSPVVVASSMGLVALGGKSSTSSQVKTV    86
LocHSP47_1   46    SLAFNLYHNMAKDKNA--ENILFSPVVVASSLGLVHLGGKSSTASQAKTV    93
DreHSP47_1   36    NLAFNLYHNVAKEKGL--ENILISPVVVASSLGMVAMGSKSSTASQVKSV    83
GacHSP47_1   36    NLAFSLYHNMAKDKDT--ENILLSPVVVASSLGMVALGGKASTASQVKTV    83
GmoHSP47_1   36    DLAFSLYHNMALDKGT-GDNILVSPVVVASSLGVLALGGKAATASQVKTV    84
OlaHSP47_1   36    NLAFSLYHSMAKDKST--DNIAISPVVVASSLGMVALGGKASTASQVKTV    83
OniHSP47_1   36    NLAFSLYHTMAKEKDT--ENIIISPVVVASSLGLVALGGKASTASQVKTV    83
PfoHSP47_1   36    NLAFSLYHKMAQDKNT--ENIVVSPVVVASSLGLVALGGKASTASQVKTV    83
TruHSP47_1   48    NLAFSLYHNMAKDKNV--ENILISPVVLASSLGMVALGGKASTASQVKTV    95
XmaHSP47_1   36    NLAFSLYHKMAQDKNT--DNIVVSPVVVASSLGLVALGGKATTASQVKTV    83
DreHSP47_2   34    TLAFNLYQNMAKDKDI--ENILISPVVVASSLGLVALGGKSNTASQVKTV    81
OniHSP47_2   41    NLAFSLYQSMVKDKNV--ENIIISPVVVASSLGLVALGGKASTASQVKTI    88
GmoHSP47_2   41    SLAFSLYQRVAQEKDT--ENILISPVVVASALGLVALGGKASTASQVKSL    88
XmaHSP47_2   41    NLAISLYQNMAKEKGA--ENILISPVVVASSLGLVALGGKGSTASQVKTI    88
GacHSP47_2   41    KLAFSLYQNMAKEKSV--ENILISPVVVASTLGLVALGGKAATSSQVKTL    88
PfoHSP47_2   41    NLAISLYQNVAKEKGA--ENILISPVVVASSLGLVALGGKGSTASQVKTI    88
AmeHSP47_2   34    NLAFDLYHNMAKEKDM--ENILISPVVVASSLGVVALGGKSNTATQVKTV    81
LocHSP47_2   36    NLGLNLYHTMVKDPSLKSENILFSPVVVATSLGVMSLGAKDKTASQVKSL    85
AmeHSP47_3   49    SLGLQLYRALRKEGSN--TNTLISPALLASSLSALSRTAKGTTAEQLQEL    96
DreHSP47_3   45    SLGLQLYRSLRTNGSQ--TNTFISPLLLANSLLALGGGAKGSTASQFHDL    92
OniHSP47_3   54    TLGLRLYQALRSDSTS--VNVLFSPLLVASSLGALGEGSGGNTSNQVQGL   101
OlaHSP47_2    7    ALGLRLYRALRTPSGS--VNTLFSPLLLASSLKALSEASSGKTSTQIQAL    54
TruHSP47_2   51    ALGLRLYQALRSDSRS--VNTLFSPLLAASSLGALGGGSAGASASQFQDL    98
TniHSP47      -    --------------------------------------------------     -
XmaHSP47_3   47    PLGVRLYQALRSDPSS--VNTLFSPLLLASSLEALSEGSAGKTAEQIREL    94
LocHSP47_3   35    ALGLRLYRALRNGTQP-PENPLLSPLLLAASLGALSQGAGGATAQQIRGL    83
PfoHSP47_3   43    PLGVRLYQALRSDPGS--VNTLFSPLLLASSLEALSEGSAGKTAEQM---    87
PfoHSP47_4   50    PLGVRLYQALRSDPGS--VNTLFSPLLLASSLEALSEGSAGKTAEQM---    94
PmaHSP47     56    AFAIDLYQSVAKAVPA-MENVVLSPVLVASALGAAQLGASSGTASRLLKA   104
LjaHSP47     91    AFAIDLYQSVAKAVPA-TENVVLSPVLVASALGAAQLGASSATASRLLKA   139
                   ~~~hA~~~~~~~~  =s6B=>    ~~~~~~hB~~~~  ~~~~~hC~~~~
```

```
                      #                    102c
                                            |  @@@           @@@
HsaHSP47      97    LSAEQL--RDEEVHAGLGELLRSLSNSTA---------RNVTWKLGSRLY   135
MmuHSP47      96    LSAEKL--RDEEVHTGLGELLRSLSNSTA---------RNVTWKLGSRLY   134
RnoHSP47      96    LSAEKL--RDEEVHTGLGELLRSLSNSTA---------RNVTWKLGSRLY   134
GgaHSP47      84    LSADKL--NDDYVHSGLSELLNEVSNSTA---------RNVTWKIGNRLY   122
TguHSP47      84    LSADKL--NDDYVHSGLSELLSEVSNSTA---------RNVTWKIGNRLY   122
MgaHSP47      97    LSADKL--NDDYVHSGLSELLNEVSNSTA---------RNVTWKIGNRLY   135
PsiHSP47      84    LSADKL--NDEYVHSGLSELLSEVSNSTA---------RNVTWKLGNRLY   122
LchHSP47      84    LSVDKL--KDEHLHAGLSELLNEVSNSTA---------RNVTWKMGSRLY   122
AmeHSP47_1    87    LKADAL--KDEHLHTGLSELLSEVSDPAA---------RNVTWKINNRLY   125
LocHSP47_1    94    LSADKV--KDDQLHTGLSELLTEVSNSTA---------RNVTWKIGSRLY   132
DreHSP47_1    84    LKADAL--KDEHLHTGLSELLTEVSDPQT---------RNVTWKISNRLY   122
GacHSP47_1    84    LSADKL--KDEHLHAGLSELLSEVSDAKS---------RNTTWKINNRLY   122
GmoHSP47_1    85    LRADAL--KDEHLHAGLSELMSEVSDAKT---------RNTTWKISNRLY   123
OlaHSP47_1    84    LSADKL--KDEHLHSGLSELLSEVSDTKA---------RNTTWKINNRLY   122
OniHSP47_1    84    LSADKL--KDEHLHAGLSELLTEVSNAKT---------RNTTWKINNRLY   122
PfoHSP47_1    84    LSADKL--KDEHLHAGLSELLSEVSDQKT---------RNSTWKINNRLY   122
TruHSP47_1    96    LSADKL--KDEHLHAGLSELLTELSDADK---------RNTTWKINNRLY   134
XmaHSP47_1    84    LSADKL--KDEHLHAGLSELLSVVSNQKT---------RNSTWKINNRLY   122
DreHSP47_2    82    LSAASV--KDEQLHSGLSELLTEVSNPKA---------RNVTWKISNRFY   120
OniHSP47_2    89    LNVAKV--KDEHLHSSLAELLIEMSDPKT---------RNITWKISNRLY   127
GmoHSP47_2    89    LKADKL--KDEQLHAGLGELLSEVSDPAV---------RNVTWKIRSRLY   127
XmaHSP47_2    89    LNAAKV--QDEQLHSGLAELLTEVSDPKE---------RNVTWKISNRLY   127
GacHSP47_2    89    LNAAKV--KDEQLHSGLAELLTEVSDPKT---------RNVTWKISNRMY   127
PfoHSP47_2    89    LNAAKV--QDEQLHSGLAELLTEVSDPKE---------RNVTWKISNRLY   127
AmeHSP47_2    82    LSGNKV--KDENLHSSLAEILTEVSNSTE---------RNVTWKISNRLY   120
LocHSP47_2    86    LRVN-L--HEDKLHPAFSELFNDVSNETA---------RNTTWKVGSRLY   123
AmeHSP47_3    97    LKNAKV---EKKTEEALSDTLKSMREGNG---------TSYTLHGSSALF   134
DreHSP47_3    93    LRITK---NENVVGETLTTAQKAVHESNG---------TSYILHSSSALF   130
OniHSP47_3   102    LNPPSPVKADTQVPERLAEALKSFSEANG---------TSFHLHASSAVF   142
OlaHSP47_2    55    LRPET---PSETSDGVLSGALKRFTEADG---------SSFHLRWSSAVF    92
TruHSP47_2    99    LKASSS--AKAGAEL-LSESLKSLGKSNG---------TSFHAHASTALF   136
TniHSP47       -    --------------------------------------------------     -
XmaHSP47_3    95    LQPPSG--TKEAVGELLSMALESFVKANG---------TSFQIHASSGVF   133
LocHSP47_3    84    LGAGAM--TPDALAGALAGLLDELHARHN---------ASYALHAAGALF   122
PfoHSP47_3    88    --PPSG--TKEAVGGLLSVALESFVKANG---------TSFQIHASSCVF   124
PfoHSP47_4    95    --PPSG--TKEAVGGLLSVALESFVKANG---------TSFQIHASSCVF   131
PmaHSP47     105    INPSGL--PGEGFHSGLAEVLGDLASQEEEAAAAAATWRNHTWKAASRVY   152
LjaHSP47     140    INPSGL--PGEGFHSGLAEVLGDLASQEEEAAAAAATWRNHTWKAASRVY   187
                                 ~~~~~~~~~~hD~~~~~~~~~~~~~~~~  ===s2A==
```

```
                             #                #         ##   #    #
HsaHSP47     136   GPSSVSFADDFVRSSKQHYNCEHSKINFRDKRSALQSINEWAAQTTDG-K   184
MmuHSP47     135   GPSSVSFADDFVRSSKQHYNCEHSKINFRDKRSALQSINEWASQTTDG-K   183
RnoHSP47     135   GPSSVSFADDFVRSSKQHYNCEHSKINFRDKRSALQSINEWASQTTDG-K   183
GgaHSP47     123   GPASINFADDFVKNSKKHYNYEHSKINFRDKRSALKSINEWAAQTTDG-K   171
TguHSP47     123   GPASITFAEDFVKNSKKHYNYEHSKINFRDKRSALKSINEWAAQTTDG-K   171
MgaHSP47     136   GPASINFADDFVKNSKKHYNYEHSKINFRDKRSALKSINEWAAQTTDG-K   184
PsiHSP47     123   GPSSINFADDFVKSSKKHYNYEHSKINFRDKRSALKSINEWASQTTDG-K   171
LchHSP47     123   GPSSVTFADDFVKRSKKHYSIDHSKINFRDKRSALKSINDWAAKTTDG-K   171
AmeHSP47_1   126   GPSSVSFADEFVKSSKKHYNYEHSKINFRDKRSAINSINEWAAKSTDG-K   174
LocHSP47_1   133   GPSSVNFADEFVKSSKKHYNYEHSKINFRDKRSAVKSINEWAAQTTDG-K   181
DreHSP47_1   123   GPSSVSFAEDFVKNSKKHYNYEHSKINFRDKRSAINSINEWAAKTTDG-K   171
GacHSP47_1   123   GPSSVSFADDFVKNSKKHYNYDHSKINFRDKRSAVNSINEWAAKSTDG-K   171
GmoHSP47_1   124   GPSSVSFADAFVKDSKKRYNYDHSKINLKDKKSAVNSINEWAAASTNG-K   172
OlaHSP47_1   123   GPSTVSFADDFVKNSKKHYNYDHSKINLRDKRSAVNSINEWAAKSTDG-K   171
OniHSP47_1   123   GPSSVSFADEFVKNSKKHYNYDHSKINFRDKRSAVNSINEWAAKSTGG-K   171
PfoHSP47_1   123   GPSSVSFADDFVKTSKKHYNYDHSKINFRDKRSAVNSINEWAAKSTDG-K   171
TruHSP47_1   135   GPSSVSFSDDFVKSSKKHYKYDHSKINFRDKRSAVNSINEWAAKATDG-K   183
XmaHSP47_1   123   GPSSVSFADDFVKTSKKHYNYDHSKINFRDKRSAVNSINEWAAKSTDG-K   171
DreHSP47_2   121   GPSSVSFVDDFLKSSKKHYNYDHSKINFRDKRSAVKAINDWASKSTDG-K   169
OniHSP47_2   128   GPSSVTFVDDFVKSSKKHYNCEHSKINIRDKKSAVKSINEWAANSTDG-K   176
GmoHSP47_2   128   GPSSITFTESFLKKSKKLYNCEHSKINFKDKKSAMKAINEWGVKSTDG-K   176
XmaHSP47_2   128   GPSSVTFAENFVKSSKKHYKCDHSKINFRDKKSAVNSINEWAAKSTGG-K   176
GacHSP47_2   128   GPSSVTFVEDFIRSSKKHYNCDYSKINFHDKKSAASSINEWAAKSTDG-K   176
PfoHSP47_2   128   GPSSVTFAEDFVKSSKKHYKCDHSKINFRDKKSAINSINKWAAKSTDG-K   176
AmeHSP47_2   121   GPSSVNFVDDFLKNSKKHYKYEHSKINFRDKRSAVKAINEWGSKSTDG-K   169
LocHSP47_2   124   GPTSAELRQEFVEKSRKHYRHDHSKINFRDKRNALKSINEWAAENTGG-R   172
AmeHSP47_3   135   SKQVPTLEKSLLEELQAQFGLGHVALGAGGKEADMEKLRSWAKGGMGGLE   184
DreHSP47_3   131   SKQAPELEKSFLEKLQTHFGMQHVALEDAQKQSDMEKLQYWAKSGMDGEE   180
OniHSP47_3   143   SKQVSPVDQAFVKQSQTRFRVKHQTLGKGDSKADLKQLQDWAKATLSGLE   192
OlaHSP47_2    93   TKQAPALRQAFVKESQSKFGLQHQTLGKGDPSADLKRLGDWAKAGTGGLE   142
TruHSP47_2   137   SKEAPQVSQAFVKDSQARFGLQHQPLGKGDSKADLKRLRSWAKRATS--R   184
TniHSP47       -   --------------------------------------------------     -
XmaHSP47_3   134   TKQAPAISQAFAKEIQSRFRLQHRTMGKGDAKADLKQLREWAEAALSGKE   183
LocHSP47_3   123   TSKGQSPRQAFLKEAETRLRLQHATLSPGDKQGSLRALNAWAERATQG-A   171
PfoHSP47_3   125   TKQAPAVGQAFAKEIQSRFRLQHRTLGKGDAKADLRQLCQWAEAALGGKE   174
PfoHSP47_4   132   TKQAPAVGQAFAKEIQSRFRLQHRTLGKGDAKADLRQLCQWAEAALGGKE   181
PmaHSP47     153   APSGVTFSQGFVSSSKARYGLQHDKVNLKDKRGALKALNEWAAQNTGG-K   201
LjaHSP47     188   APSGVTFSQGFVSSSKARYGLQHDMVNLKDKRGALKALNEWAAQNTGG-K   236
                   ==>     ~~~~~hE~~~~    ==> ~~~~~~~~~hF~~~~~~~~~
                                       s1A
```

```
                                  #
                         #    #    ##192a
                                      |          #
HsaHSP47    185   LPEVTKDVERTDGALLV-NAMFFKPHWD--EKFHHKMVDNRGFMVTRSYT   231
MmuHSP47    184   LPEVTKDVERTDGALLV-NAMFFKPHWD--EKFHHKMVDNRGFMVTRSYT   230
RnoHSP47    184   LPEVTKDVERTDGALLV-NAMFFKPHWD--EKFHHKMVDNRGFMVTRSYT   230
GgaHSP47    172   LPEVTKDVEKTDGALIV-NAMFFKPHWD--EKFHHKMVDNRGFMVTRSYT   218
TguHSP47    172   LPEVTKDVEKTDGALIV-NAMFFKPHWD--EKFHHKMVDNRGFMVTRSYT   218
MgaHSP47    185   LPEVTKDVEKTDGALIV-NAMFFKPHWD--EKFHHKMVDN-------SYT   224
PsiHSP47    172   LPEVTKDVEKTDGALIV-NAMFFKPHWD--EKFHHKMVDNRGFMVTRSYT   218
LchHSP47    172   LPEITKDVEKTDGAMII-NAMFYKPHWD--EKFHEKMVDNRGFMVSRSYT   218
AmeHSP47_1  175   LPEITKDVKNTDGAMII-NAMFFKPHWD--EKFHHKMVDNRAFLVTRSYT   221
LocHSP47_1  182   LPEVTKDVEKTDGAMIV-NAMFFKPHWD--EKFHHKMVDNRGFLVSRSYT   228
DreHSP47_1  172   LPEITKDVKNTDGAMIV-NAMFFKPHWD--EKFHHKMVDNRGFLVTRSHT   218
GacHSP47_1  172   LTEITKDVQNTDGAMIV-NAMYFKPHWF--ETFNDQMVDNRGFLVTRSFT   218
GmoHSP47_1  173   LPEVTKDVPNADGAMLI-NAMFFKPHWD--EMFHESMVDSRSFMVTRSFT   219
OlaHSP47_1  172   LPEVTKEVQNPDGAMIV-NAMFFKPHWE--ERFHEKMVDTRGFLVTRSFT   218
OniHSP47_1  172   LPEVTKDVQNPDGAMIV-NAMFFKPHWE--EKFHDKMVDTRGFLVTRSFT   218
PfoHSP47_1  172   LPEITKDVQNPDGAMIV-NAMFFKPHWD--ERFHEKMVDNRGFLVTRSFT   218
TruHSP47_1  184   LPEITKDVQNADGAMIV-NAMFFKPHWD--ERFHDKMVDTRGFLVTRSHT   230
XmaHSP47_1  172   LPEITKDVQNPDGAMIV-NAMFFKPHWD--ERFHEKMVDNRGFLVTRSFT   218
DreHSP47_2  170   LPEVTKDVEKTDGAMII-NAMFYKPHWN--EQFHHKMVDNRGFLVHRSFT   216
OniHSP47_2  177   LPEVTKNVEKTDGAMII-SAMFFKCHWD--EQFHREMVDNRGFMVSRSYT   223
GmoHSP47_2  177   LPEVAKNVEKTDGAMII-NAMFFKPHWN--EQFHPKMVDGRVFLASRGLT   223
XmaHSP47_2  177   LPEVTKDVENTDGAMII-NAMFFKPHWD--EQFHPTMVDNRGFLVSGSYT   223
GacHSP47_2  177   LPEVTKVVEQTDGAMIV-NAMFFKPHWD--EQFHHQMVDNRGFMLPRSYT   223
PfoHSP47_2  177   LPEVTKDVENTDGAMII-NAMFFKPHWD--EQFHPTMVDNRGFLVSRSYT   223
AmeHSP47_2  170   LPEITKDVEKTDGAMII-NAMFYKPHWD--QQFHHKMVDNRGFLVHRSFT   216
LocHSP47_2  173   LSEITRDLPGSDGAMFV-NAMHFKPHWG--EAFHHKMVDNRGFLMSKTHS   219
AmeHSP47_3  185   GSLLKEGPDAKDGAVILANALHFKGLWD--RSFETENQDLRTFLGTKY--   230
DreHSP47_3  181   TAALKTALESKAGALILANALHFKGLWD--RGFYHENQDVRSFLGTKY--   226
OniHSP47_3  193   GTPLEAEIKARAGALILANAVRLKGLWE--REFSEESTDRRTFLGKKY--   238
OlaHSP47_2  143   GAPLEGGIKAKAGAVILANALHFKGLFDLLMNYYHIFKAVSAYF--EFFI   190
TruHSP47_2  185   RNELSSDAYRSSQLLLFPPAEFKPGLWE--REFGEGSSDLRTFLGKKY--   230
TniHSP47      -   --------------------------------------------------     -
XmaHSP47_3  184   MAPLEAEIKAKAGAMILTNALHFKGLWQ--RELSEESTDRRTFLGKKY--   229
LocHSP47_3  172   VLEVAKEMPASPGALLV-NALHFKGLWD--REFDVEGIDFRRFLGSKY--   216
PfoHSP47_3  175   GAPLEAEIKAKAGAMVLTNALHFKGLWQ--RELSEESADRRTFLGKKY--   220
PfoHSP47_4  182   GAPLEAEIKAKAGAMVLTNALHFKGLWQ--RELSEESADRRTFLGKKY--   227
PmaHSP47    202   VKEVAKELDGADGAVFV-NALFFKGRWN--EKFHHQMVDTRGFLTSRSHT   248
LjaHSP47    237   VKEVAKELNGADGAVFV-NALFFKGRWN--EKFHHQMVDTRGFLTSRSHT   283
                     =========s3A========>         ~~~~ =s4C=>   ==
                                                    hF1
```

Motif 1

```
                  #   ##    225a                     #          ##
                            |
HsaHSP47    232   VGVMMMHRT-GLYNYYDDEKEKLQIVEMPLAHKLSSLIILMPHHVEPLER   280
MmuHSP47    231   VGVTMMHRT-GLYNYYDDEKEKLQMVEMPLAHKLSSLIILMPHHVEPLER   279
RnoHSP47    231   VGVTMMHRT-GLYNYYDDEKEKLQLVEMPLAHKLSSLIILMPHHVEPLER   279
GgaHSP47    219   VGVPMMHRT-GLYNYYDDEAEKLQVVEMPLAHKLSSMIFIMPNHVEPLER   267
TguHSP47    219   VGVPMMHRT-GLYNYYDDEAEKLQVVEMPLAHKLSSMIFIMPNHVEPLER   267
MgaHSP47    225   VGVPMMHRT-GLYNYYDDEAEKLQVVEMPLAHKLSSMIFIMPNHVEPLER   273
PsiHSP47    219   VGVPMMHRT-GLYNYFDDETEKLQIVEMPLAHKLSSMIFIMPNHVEPLER   267
LchHSP47    219   VAVPMMHRT-GLYHFYDDEVNKLQVLEMPLAHKLSSMLFVMPYHVEQLDR   267
AmeHSP47_1  222   VSVPMMHRT-GLYPFHEDTENRLFVLNMPLAHKKSSVIFIMPYHVEPLER   270
LocHSP47_1  229   VSVPMMHRT-GLYMFHEDSEKRLYVLSMPLAHKMSSMIIIMPYHVEPLER   277
DreHSP47_1  219   VSVPMMHRT-GIYGFYEDTENRFLIVSMPLAHKKSSMIFIMPYHVEPLDR   267
GacHSP47_1  219   VGVPMMHRT-GLYDFHEDKENRLFVLNMPLGKKEASMILIMPYHLEPLAR   267
GmoHSP47_1  220   VGIPMMHRT-GLYDFHEDTENRLFALNMPLGKNQASMILIMPYYLEPLER   268
OlaHSP47_1  219   VGVSMMHRT-GLYDFFEDTENKIFVLNMPLGQKQASMILIMPYHLEPLER   267
OniHSP47_1  219   VGVPMMHRT-GLYDFYEDTENKLFVLNMPLGQKQASMILIMPYHLEPLER   267
PfoHSP47_1  219   VGVSMMHRT-GLYDFYEDKENNLYILNMPLGKKQASMILIMPYHLEPLER   267
TruHSP47_1  231   IGISMMHRT-GLYDFYDDEVNRIYVLNMPLGQKQASMILIMPYHLEPLER   279
XmaHSP47_1  219   VGVSMMHRT-GLYDFYEDKENNLYILNMPLGKKEASMILIMPYHLEPLDR   267
DreHSP47_2  217   VSVPMMHRT-GIYGFLDDTTNKLLVLEMPLAHKMSSLVLIMPYHVESLER   265
OniHSP47_2  224   VSVQMMHRT-GLFGFYDDKDNKLSILSMPLSHKKSTVVFIMPYHVEPLDR   272
GmoHSP47_2  224   ISVEMMHRT-GLYDYYEDVDNKLTVLSIPLAHKKSSVVFVMPLHVEPLER   272
XmaHSP47_2  224   VALQMMHRT-GLFDFYDDTTNKLYLLSMPLAHKKSTVVFVMPHHMEPLDR   272
GacHSP47_2  224   VSVQMMHRT-GLYGFYDDKTNKLTLLSMPLAHKKSTVVVIMPHQVEPLQR   272
PfoHSP47_2  224   VALQMMHRTVGLFDFYDDTTNKLYLLSLPLAHKKSTVVFIMPYHMEPLDR   273
AmeHSP47_2  217   VSVPMMHRT-GIYGFLEDTTNNLFVLEMPLAHKMSSVVFIMTYHVEPLER   265
LocHSP47_2  220   VSVPMMHRT-GFYKYYEDEVNQLQIVEMPLGHRQTSMMIIMPFHVEPLER   268
AmeHSP47_3  231   TKVMMMHRA-GVYRHYEDMENMVQVIELGLWSGKASMLLILPFHVESLAR   279
DreHSP47_3  227   TKVPMMHRS-GVYRHYEDMENMVQVLELGLWEGKASIVLLLPFHVESLAR   275
OniHSP47_3  239   TKVVMMHRA-GLYRFHEDTDNMVQVLEAPLWGGRASVVLLLPFHVENLAR   287
OlaHSP47_2  191   EGKSLFSPLTGLFRFHEDMENMVQVLEVPLWGGQASAVFLMPFHVENLAR   240
TruHSP47_2  231   TKIMMMHRA-GLYRFHEDIQNMVQVLEAPLWGGKASMVLLLPFHVEDLAR   279
TniHSP47      -   --------------------------------------------------     -
XmaHSP47_3  230   TKVIMMHRA-GRYRFHEDVDNMVQVLEAPLWGGKASLVLLLPFHVENLAR   278
LocHSP47_3  217   TKVAMMHRA-GIYRHYEDLEKMVQILEMPLAGGSASMVLLLPLHVEPLSR   265
PfoHSP47_3  221   TKVTMMHRA-SRYRFHEDVDNMVQVLEAPLWGGRASLVLLLPFHVENLAR   269
PfoHSP47_4  228   TKVTMMHRA-GRYRFHEDVDNMVQVLEAPLWGGRASLVLLLPFHVENLAR   276
PmaHSP47    249   ISIQMMHRT-GFYNFYHDEKAQVQLLEMQLKGNLESLLIALPLHTESLER   297
LjaHSP47    284   ISIQMMHRT-GFYNFYHDEKAQVQLLEMQLKGSLESLLIVLPLHTESLER   332
                  ====s3C======>   s1B>   ==s2B=>   ==s3B==>      ~~~
```

```
                                                        300c
                                             ##         #   #   #
HsaHSP47    281   LEKLLTKEQLKIWMGKMQKKAVAISLPKGVVEVT--HDLQKHLAGLGLTE    328
MmuHSP47    280   LEKLLTKEQLKAWMGKMQKKAVAISLPKGVVEVT--HDLQKHLAGLGLTE    327
RnoHSP47    280   LEKLLTKEQLKTWMGKMQKKAVAISLPKGVVEVT--HDLQKHLAGLGLTE    327
GgaHSP47    268   VEKLLNREQLKTWASKMKKRSVAISLPKVVLEVS--HDLQKHLADLGLTE    315
TguHSP47    268   VEKLLNKEQLKTWTGKMKKRSVAISLPKVVLEVS--HDLQKHLGGLGLTE    315
MgaHSP47    274   VEKLLNREQLKTWAGKMKKRSVAISLPKVVLEVS--HDLQKHLADLGLTE    321
PsiHSP47    268   VEKLLTREQLKTWIGKLKKRAVAISLPKVSLEVS--HDLQKHLADLGLTE    315
LchHSP47    268   LEKLLTREQVNTWSSKLQQVAVAVSLPKVSMEVS--HELQKYLATLGLTE    315
AmeHSP47_1  271   LEKLLTRQQLDKWISKMEERAVAVSLPKVSMEVS--HDLQKHLGELGLTE    318
LocHSP47_1  278   LEKLLTRKQLDTWISKMEERAVAVSLPKVCMEVS--HNLQKHLGELGLTE    325
DreHSP47_1  268   LENLLTRQQLDTWISKLEERAVAISLPKVSMEVS--HDLQKHLGELGLTE    315
GacHSP47_1  268   LEKLLTRKQVDTWISKMENIAVAISLPKIALEVS--HNLQKHLAELGLTE    315
GmoHSP47_1  269   LEKLLTREQVDTWVGKMEKRAVAVSMPKVSMEVS--HNLQKHLATLGLTE    316
OlaHSP47_1  268   LEKLLTRKQVDTWLSKMENRAVAISLPKVSVEVS--HNLQKYLAELGLTE    315
OniHSP47_1  268   LEKLLTRKQVDTWLSKMENRAVAISLPKISVEVS--HNLQKHLAELGLTE    315
PfoHSP47_1  268   LEKLLTKKQVDTWVSKMENRAVAISLPKISLEVS--HNLQKYLAELGLTE    315
TruHSP47_1  280   LEKLLSKKQVDTWISKMTNKAVAISLPKISVDVS--HNIQKYLSELGLTE    327
XmaHSP47_1  268   LEKLLTKKQVDTWLSKMENKAVAISLPKVSLEVS--HNLQKYLAELGLTE    315
DreHSP47_2  266   VEKLLTRQQLNTWVSAMEQKAVAISLPKVSMEVS--HNLQKHLAELGLTE    313
OniHSP47_2  273   LEKMLTKKQVDRWIGLLKETAIAVSLPKVSMEVS--HDLQKHLQELGLTE    320
GmoHSP47_2  273   VEKLLSGKQLDTWVAKMEEKAVAVSLPKISMEVS--HNLQKHLGELGLTD    320
XmaHSP47_2  273   VEKMLSKKQLDTWMGKMKQTAVAVSLPKVSMEVS--HNLQKHLQDLGLTE    320
GacHSP47_2  273   LEKMLTRKQLDTWMGKLQQTAVAVSLPKVTMEVS--HNLQKHLGKLGLTE    320
PfoHSP47_2  274   LEKMLSKKQLDTWMGKMKQTAVAVSLPKVSMEVS--HNLQKHLQDLGLTE    321
AmeHSP47_2  266   VEKLLTRKQVETWLSKLEQKAVAVSLPKVSMEVS--HNLQKHLGELGLTE    313
LocHSP47_2  269   LEKMLTKEQLSVWSNKLQERAVAVSLPKINVDVN--HELQKHLQELGLAE    316
AmeHSP47_3  280   VDRLLTLQQLETWMGKLSSMSMALSLPQVNISST--LSLQKQLAVLGVAD    327
DreHSP47_3  276   LDRLLTLDRLEKWFGKLNSTSMALSLPRTKMSSV--VNLQKQLAAMGLVD    323
OniHSP47_3  288   LEKLLTLELLSKWLEKASVTSVAISLPKANITST--LSLQKQLVTLGLTD    335
OlaHSP47_2  241   LDKLLSLDLL-SWLERTSVTSVAISMPKANITST--LSLQKALSGLGLMD    287
TruHSP47_2  280   LDKLLTVQLVSKWLEKSSMSSISISLPKANISSA--LSLQKPLSALGLVD    327
TniHSP47      1   --------------------------------------LQKPLSALGVTD     12
XmaHSP47_3  279   LDKLLTPELLSKWLERTAVTGVALSLPKANITSS--LSLQKQLSSLGLSD    326
LocHSP47_3  266   LEGLLTQEQLTVWLGRLANRSIGVSLPRAIFKIQSLSDFQKQLAALGMAD    315
PfoHSP47_3  270   LDKLLTPELLSKWLERTAVTGVALSLPKANISSS--LSLQKQLTSLGLSD    317
PfoHSP47_4  277   LDKLLTPELLSKWLERTAVTGVALSLPKANISSS--LSL-----------    313
PmaHSP47    298   LEKLLTKQQLEEWTSKLQKKTIAVSMPKGLLQGS--ADIQNSLADLGLAE    345
LjaHSP47    333   LEKLLTKQQLEEWTSKLQKKTIAVSMPKGLLQGS--ADIKNSLADLGLAE    380
                  ~hG~~ ~~~hH~~~    ====s2C==> ====s6A===> ~~~hI~~ ~~
                                                                   hI1
```

```
                   #    #            #      #       #  #  #
HsaHSP47    329   AIDKNKADLSRMSGKKD--LYLASVFHATAFELDTD-GNPFDQDIYGREE   375
MmuHSP47    328   AIDKNKADLSRMSGKKD--LYLASVFHATAFEWDTE-GNPFDQDIYGREE   374
RnoHSP47    328   AIDKNKADLSRMSGKKD--LYLASVFHATAFEWDTE-GNPFDQDIYGREE   374
GgaHSP47    316   AIDKTKADLSKISGKKD--LYLSNVFHAAALEWDTD-GNPYDADIYGREE   362
TguHSP47    316   AIDKTKADLSKISGKKD--LYLSNVFHAAALEWDTE-GNPYDADIYGREE   362
MgaHSP47    322   AIDKTKADLSKISGKKD--LYLSNVFHAAALEWDTD-GNPYDADIYGREE   368
PsiHSP47    316   AIDKNKADLSKISGKKD--LYLSNVFHAAALEWDTE-GNPFDADIYGREE   362
LchHSP47    316   AVDKAKADLSTISGKKD--LYLANVFHAAALEWDTE-GNPFDSSIYSREE   362
AmeHSP47_1  319   VVDKSKADLSNISGKKD--LYLSNVFHASAWEWDTE-GNPFDTSIFGSDK   365
LocHSP47_1  326   AVDKAKADLSNISGKKD--LYLSNVFHASAMELDTE-GNPFDTNIYGSEK   372
DreHSP47_1  316   AVDKSKADLSNISGKKD--LYLSNVFHASSLEWDTE-GNPFDPSIFGSEK   362
GacHSP47_1  316   AVDKSKADLSNISGKKD--LYLSNVFHASALELDVK-GNPFDTSVYGSGK   362
GmoHSP47_1  317   AVDKAKADLSNITGKKN--LYLSNVFHASALELDTL-GNPFDTSIFGSEK   363
OlaHSP47_1  316   AVDKSKADLSNISGKKD--LYLSNLFHASALELDVD-GNPYDTSIFGTDK   362
OniHSP47_1  316   AVDKAKADLSNISGKKD--LYLSSVFHASALELDIE-GNPYDTSIFGTEK   362
PfoHSP47_1  316   AVDKSKADLSNISGKKD--LYLSNVFHASALELDVD-GNPYDTSIFGTEK   362
TruHSP47_1  328   AVDKAKADLSNISGKKD--LYLSNVFHASAVELDVD-GNPYDTSIFGTEK   374
XmaHSP47_1  316   AVDKSKADLSNISGKKD--LYLSNVFHASALELDVD-GNPYDTSIFGTDK   362
DreHSP47_2  314   AVDKAKADLSNISGKKD--LYLSNVFHASAMEWDTE-GNPPDTSIFGTDQ   360
OniHSP47_2  321   AVDKSKADFSNISGKKD--LYLSSVFHASALEWDTD-GNEIDTSIFGTDK   367
GmoHSP47_2  321   AVDKAKADLSNISGKKD--LYLANVFHASAVEWATE-GNPIDPTVYSSDK   367
XmaHSP47_2  321   AVDKSKADLSNVSGKKD--LYLSGVLHAAALEWDTH-GNEIDTSIFGTEK   367
GacHSP47_2  321   AVDKSKADLSKISGKKD--LYLASVFHASAMEWDTD-GNEMDLSLFGTDK   367
PfoHSP47_2  322   AVDKSKADLSNVSGKKD--LYLSGVLHAAALEWDTH-GNEIDTSIFGTEK   368
AmeHSP47_2  314   AVDKTKADLSNISGKKE--LYLSNVFHASALEWDTE-GNPPDTSVFGSDK   360
LocHSP47_2  317   AVDKSKADFSGMTGKKD--LHLSNFLHATAFDLDTD-GNPFDQDIYGREE   363
AmeHSP47_3  328   AWDQKVADFSGLSSKGKGKLHLGGVLHWASLELASESGSKDGSN--EDEH   375
DreHSP47_3  324   AWNETSADFSTLSSLGRGKLHLGAVLHWTTLELAPESGSKDDVL--EDED   371
OniHSP47_3  336   AWDQKLADFSGVSAKSKGKLHLGGVLHWATLELAAQAGKEDADL--QEEH   383
OlaHSP47_2  288   AWDQKVADFSGVSDKSKGKLHLGAVLHWASMELAPQAGEGEVDV---EEN   334
TruHSP47_2  328   AWDQKVADFSGVSGKAKGKLHLSAVLQWTSLELAAQAGPGEDQL--EEEE   375
TniHSP47     13   AWDQKVADFSGVSGKSEGKLHLGAVLQWTSLELAAQAGPGEEEL--EEEK    60
XmaHSP47_3  327   AWDQKAADFSGMSDKSKGKLHLGGALHWASLELDPAAGEQDVDL--EEEN   374
LocHSP47_3  316   AWDGARADFAGVTGQKG--LRLAAVLHAAALELGPE-GAPVQQ---EEE-   358
PfoHSP47_3  318   AWDQKAADFSGMSDKSKGKLHFGGALHWASLELGPTAGKEDADL--EEEN   365
PfoHSP47_4    -   --------------------------------------------------     -
PmaHSP47    346   VGDKAKADFSGMTGGRE--LHLGSLLHTAALEFDTE-GEEYDMSVHGHPD   392
LjaHSP47    381   VGDKAKADFSGMTGGRE--LHLGSLLHAAALEFDTE-GEEYDMSVHGHPD   427
                  ~                      ===s5A==> ==========s4A====
```

Motif 2

```
                                 ##          ## #    #
HsaHSP47     376   LRSPKLFYADHPFIFLVRDTQSGSLLFIGRLVRPKGDKMRDEL   418
MmuHSP47     375   LRSPKLFYADHPFIFLVRDNQSGSLLFIGRLVRPKGDKMRDEL   417
RnoHSP47     375   LRSPKLFYADHPFIFLVRDNQSGSLLFIGRLVRPKGDKMRDEL   417
GgaHSP47     363   MRNPKLFYADHPFIFMIKDSKTNSILFIGRLVRPKGDKMRDEL   405
TguHSP47     363   MRNPKLFYADHPFIFMIKDTKTNSILFIGRLVRPKGDKMRDEL   405
MgaHSP47     369   MRNPKLFYADHPFIFMIKDSKTNSILFIGRLVRPKGDKMRDEL   411
PsiHSP47     363   MRNPRLFYADHPFVFVIKDNKTNSILFIGRLVRPKGDKMRDEL   405
LchHSP47     363   MRNPKLFYVDHPFVFLIKDNKTNSILFIGRLVKPKGEKMRDEL   405
AmeHSP47_1   366   LRNPKLFYADHPFIFVVKDNQTNSILYIGRLVKPKGDKMRDEL   408
LocHSP47_1   373   LRNPKLFYADHPFIFVIKDNKTNSILYIGRLVRPKGDKMRDEL   415
DreHSP47_1   363   MRNPKLFYADHPFIFLVKDNKTNSILFIGRLVRPKGDKMRDEL   405
GacHSP47_1   363   LTNPRLFYADHPFVFLVKDNKTNSILYIGRVVKPKGDKMRDEL   405
GmoHSP47_1   364   LRNPRLFYVDHPFIFLVKDNKSGAIMYIGRMVKPNGDKMRDEL   406
OlaHSP47_1   363   LRNPKLFYVDHPFIFLVKDNKTNSILYIGRVVRPKGEKMRDEL   405
OniHSP47_1   363   LRNPKLFYVDHPFIFLVKDNKTNSILYIGRVVKPKGDKMRDEL   405
PfoHSP47_1   363   LRNPKLFYVDHPFIFLVKDNKTNSILYIGRVVRPKGDKMRDEL   405
TruHSP47_1   375   LKNPKLFYVDHPFIFLVKDNKTNSIMYIGRVVKPKGDKMRDEL   417
XmaHSP47_1   363   LKNPKLFYVDHPFIFLVKDNKTNSILYIGRVVRPKGDKMRDEL   405
DreHSP47_2   361   LKNPKLFYADHPFVFLVKDNKTNSILFMGRLIRPKGDKMRDEL   403
OniHSP47_2   368   LKNPKLFYADHPFIFLVKDQKTSSILFIGRMVRPKGEKMRDEL   410
GmoHSP47_2   368   LKNPKLFYADHPFLFLVKDLKTKSILYMGRMVRPKGDKIRDEL   410
XmaHSP47_2   368   LKNPRLFYADHPFIFLIKDKKTNSLLFIGRMVRPKGEKMRDEL   410
GacHSP47_2   368   LKDPKLFYADHPFIFLVKDQKTNSILFIGRMVRPKGDKMKDEL   410
PfoHSP47_2   369   LKTPKLFYADHPFVFFIKDKKTNSLLFIGRMVRPKGEKMRDEL   411
AmeHSP47_2   361   LKNPKLFYADHPFIFLVKDNKTKSILYIGRLVRPKGDKMRDEL   403
LocHSP47_2   364   MRSPKLFYVDHPFIFLIQDKKTNSILLIGRLVKPNGDGNHDEL   406
AmeHSP47_3   376   LDKPKLFYADHSFVILVKENSTGALVLLGALDMAEGAALHDEL   418
DreHSP47_3   372   VKKPKIFYADHSFIILVRDNSTGALLMIGALDHTDGPAIHDEL   414
OniHSP47_3   384   IDRPKLFYADHPFILFVRDSATGALLLMGALDHAEGEPLHDEL   426
OlaHSP47_2   335   VGRPKLFYADHPFLVLVRDNATGALLLMGALDHAEGELLHDEL   377
TruHSP47_2   376   IEKPKMFYADHPFVFLVRDNATGALLLMGALDHVEGEAVHDEL   418
TniHSP47      61   IEAPKLFYADHPFVFLVRDNATGALLLMGALDHVEGEAVHDEL   103
XmaHSP47_3   375   IERPKLFYADHPFIICVRDNTTGALLLMGALDNVEGDLLHDEL   417
LocHSP47_3   359   LQNTRLFYADHPFVLLVRDNQTGALLLMGALDNAQGSALHDEL   401
PfoHSP47_3   366   TERPKLFYADHPFIVFVRDDTTGALLLMGALDNVEGELLHDEL   408
PfoHSP47_4   314   -------------------QVGAPMLAA---------------   322
PmaHSP47     393   MRNPHLFYIDHPFFFLVRDARSGATLLIGRCMRPMGSGRHDEL   435
LjaHSP47     428   MRNPHLFYVDHPFFFLVRDARSGATLLIGRCMRPVGSGRHDEL   470
                   ===> ==>     ==s4B==>    ====s5B==>
                        s1C
```

Motif 3

Fig S3. Summary of sequence analyses of different HSP47/SERPINH1.

A. Set I-III of HSP47/SERPINH1 shares different sequence identity ranges with HsaHSP47/SERPINH1. It hints for recent duplication of sets I and II being very close and set III being diverged.

B. Sequence logo of the reactive center loops of three sets of HSP47/SERPINH1 depicts non-inhibitory nature and differences with loss of two residues in the set III.

C. The C-terminal ends of different HSP47/SERPINH1 proteins depicts that ER-retention signal has different in the set III with R being replaced by H.

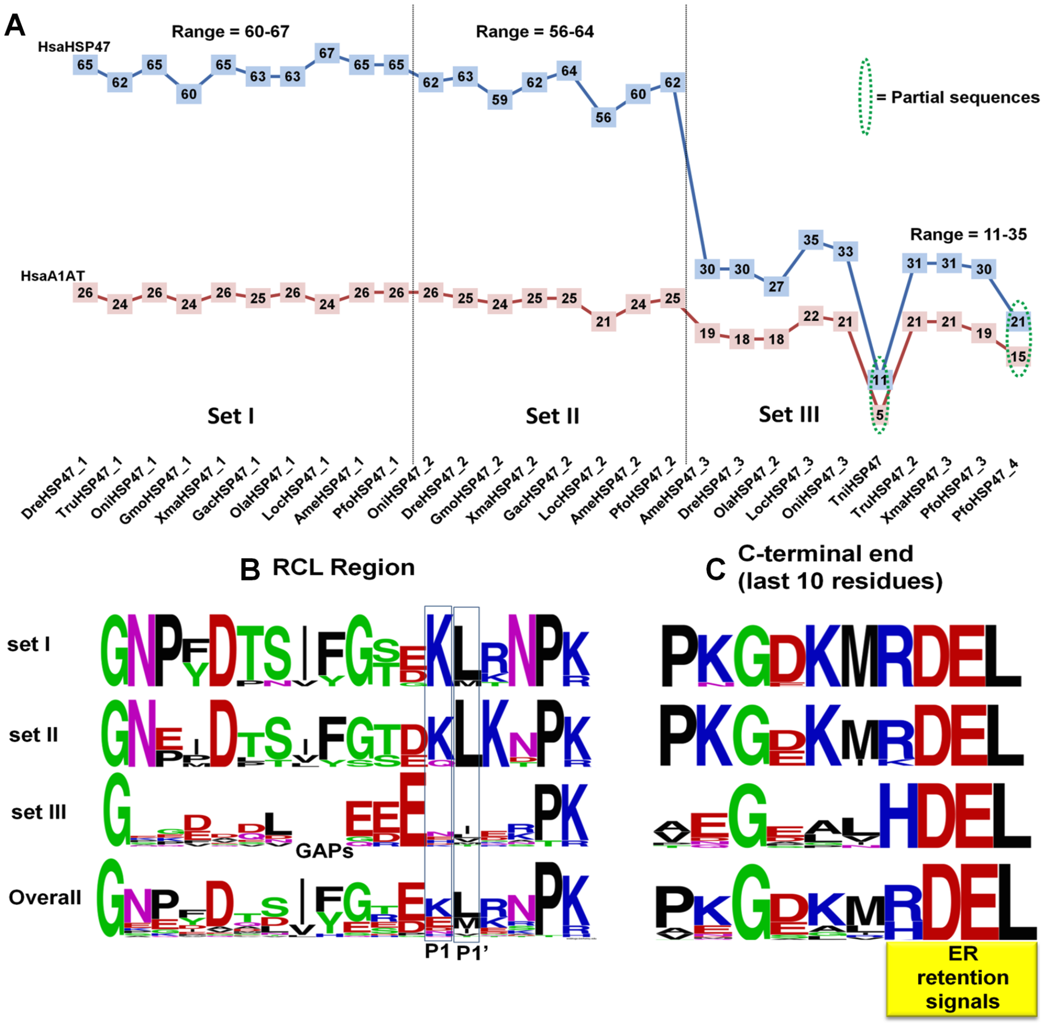

Table S1. Overview of HSP47 genes from 61 vertebrate genomes, their assemblies and genomic localization.

Species	Genome assembly	Accession id	Genomic localization
Ailuropoda melanoleuca	ailMel1 (GCA_000004335.1)	ENSAMEG00000017004	Scaffold GL192425.1: 1,119,608-1,124,040
Anas platyrhynchos	BGI_duck_1.0 (GCA_000355885.1)	ENSAPLG00000014348	Scaffold KB743381.1: 113,502-116,434
Anolis carolinensis	AnoCar2.0 (GCA_000090745.1)	ENSACAG00000002229	Scaffold GL343298.1: 714,401-718,931
Astyanax mexicanus	AstMex102 (GCA_000372685.1)	ENSAMXG00000000056	Scaffold KB871604.1: 223,363-229,678
Astyanax mexicanus	AstMex102 (GCA_000372685.1)	ENSAMXG00000006432	Scaffold KB882085.1: 1,768,034-1,776,194
Astyanax mexicanus	AstMex102 (GCA_000372685.1)	ENSAMXG00000003269	Scaffold KB882131.1: 359,489-395,080
Bos taurus	UMD3.1 (GCA_000003055.3)	ENSBTAG00000001027	Chromosome 15: 55,514,945-55,525,175
Callithrix jacchus	C_jacchus3.2.1 (GCA_000004665.1)	ENSCJAG00000015377	Chromosome 11: 63,119,525-63,130,780
Canis lupus familiaris	CanFam3.1 (GCA_000002285.2)	ENSCAFG00000005386	Chromosome 21: 23,033,455-23,042,446
Cavia porcellus	cavPor3	ENSCPOG00000022994	scaffold_103: 114,481-117,787
Chlorocebus sabaeus	ChlSab1.1 (GCA_000409795.2)	ENSCSAG00000003909	Chromosome 1: 66,788,240-66,799,072
Danio rerio	Zv9 (GCA_000002035.2)	ENSDARG00000019949	Chromosome 15: 28,732,298-28,736,597
Danio rerio	Zv9 (GCA_000002035.2)	ENSDARG00000075954	Chromosome 10: 39,878,541-39,887,981
Danio rerio	Zv9 (GCA_000002035.2)	ENSDARG00000036445	Chromosome 7: 22,441,345-22,478,600
Dasypus novemcinctus	Dasnov3.0 (GCA_000208655.2)	ENSDNOG00000019034	Scaffold JH568609.1: 111,384-121,357
Dipodomys ordii	dipOrd1	ENSDORG00000002978	GeneScaffold_4955: 160,144-165,399
Echinops telfairi	TENREC	ENSETEG00000017520	scaffold_303682: 3,151-7,601
Equus caballus	Equ Cab 2 (GCA_000002305.1)	ENSECAG00000004871	Chromosome 7: 68,605,995-68,611,500
Erinaceus europaeus	eriEur1	ENSEEUG00000003946	GeneScaffold_511: 19,319-22,838
Felis catus	Felis_catus_6.2 (GCA_000181335.2)	ENSFCAG00000011944	Chromosome D1: 59,000,253-59,009,974
Ficedula albicollis	FicAlb_1.4 (GCA_000247815.1)	ENSFALG00000006601	Scaffold JH603425.1: 18,154-20,583
Gadus morhua	gadMor1 (GCA_000231765.1)	ENSGMOG00000001309	GeneScaffold_1352: 269,125-271,324
Gadus morhua	gadMor1 (GCA_000231765.1)	ENSGMOG00000007382	GeneScaffold_4231: 9,838-13,332
Gallus gallus	Galgal4 (GCA_000002315.2)	ENSGALG00000011214	Chromosome 1: 194,288,921-194,295,617
Gasterosteus aculeatus	BROADS1	ENSGACG00000006375	groupI: 3,124,210-3,127,693
Gasterosteus aculeatus	BROADS1	ENSGACG00000020152	groupVII: 12,211,634-12,215,855
Gorilla gorilla gorilla	gorGor3.1 (GCA_000151905.1)	ENSGGOG00000005397	Chromosome 11: 72,594,231-72,604,934
Homo sapiens	GRCh37 (GCA_000001405.13)	ENSG00000149257	Chromosome 11: 75,273,101-75,283,828
Ictidomys tridecemlineatus	spetri2 (GCA_000236235.1)	ENSSTOG00000027738	Scaffold JH393308.1: 4,265,158-4,350,937
Latimeria chalumnae	LatCha1 (GCA_000225785.1)	ENSLACG00000006746	Scaffold JH129193.1: 234,078-249,245

Poecilia formosa	Poecilia_formosa-5.1.2 (GCA_000485575.1)	ENSPFOG00000013710	Scaffold AYCK01029354.1: 5,404-12,214
Pongo abelii	PPYG2 (GCA_000001545.1)	ENSPPYG00000003691	Chromosome 11: 71,005,958-71,015,006
Procavia capensis	proCap1	ENSPCAG00000010663	GeneScaffold_4297: 70,366-73,898
Pteropus vampyrus	pteVam1	ENSPVAG00000011278	GeneScaffold_1708: 259,301-263,374
Rattus norvegicus	Rnor_5.0 (GCA_000001895.3)	ENSRNOG00000016831	Chromosome 1: 170,503,596-170,510,775
Sarcophilus harrisii	Devil_ref v7.0 (GCA_000189315.1)	ENSSHAG00000009364	Scaffold GL849728.1: 535,612-545,598
Sus scrofa	Sscrofa10.2 (GCA_000003025.4)	ENSSSCG00000014859	Chromosome 9: 10,972,133-10,976,532
Taeniopygia guttata	taeGut3.2.4	ENSTGUG00000013481	Chromosome 1: 99,126,385-99,128,353
Takifugu rubripes	FUGU4	ENSTRUG00000001559	scaffold_131: 112,128-113,986
Takifugu rubripes	FUGU4	ENSTRUG00000004704	Scaffold_186: 231,121-242,595
Tarsius syrichta	tarSyr1	ENSTSYG00000007354	GeneScaffold_4812: 539-2,737
Tetraodon nigroviridis	TETRAODON8	ENSTNIG00000004097	Chromosome Un_random: 493,910-494,221
Tupaia belangeri	tupBel1	ENSTBEG00000001023	GeneScaffold_3565: 125,861-130,281
Tursiops truncatus	turTru1	ENSTTRG00000012009	scaffold_112285: 165,941-171,301
Xenopus tropicalis	JGI 4.2 (GCA_000004195.1)	ENSXETG00000010725	Scaffold GL172913.1: 1,301,229-1,303,075
Xiphophorus maculatus	Xipmac4.4.2 (GCA_000241075.1)	ENSXMAG00000001370	Scaffold AGAJ01049567.1: 337-4,406
Xiphophorus maculatus	Xipmac4.4.2 (GCA_000241075.1)	ENSXMAG00000014013	Scaffold JH556831.1: 757,758-762,448
Xiphophorus maculatus	Xipmac4.4.2 (GCA_000241075.1)	ENSXMAG00000013925	Scaffold JH557141.1: 97,083-122,348

*From webpage of the Japanese lamprey genome project (http://jlampreygenome.imcb.a-star.edu.sg/)

Lepisosteus oculatus	LepOcu1 (GCA_000242695.1)	ENSLOCG00000000987	Chromosome LG3: 474,771-481,123
Lepisosteus oculatus	LepOcu1 (GCA_000242695.1)	ENSLOCG00000014734	Chromosome LG7: 41,901,506-41,905,696
Lepisosteus oculatus	LepOcu1 (GCA_000242695.1)	ENSLOCG00000013424	Chromosome LG2: 58,538,944-58,547,367
Lethenteron japonicum	LetJap1.0 (APJL00000000.1)	JL8319	Scaffold00131: 1482314-1484962
Loxodonta africana	Loxafr3.0 (GCA_000001905.1)	ENSLAFG00000003780	SuperContig scaffold_79: 1,878,003-1,881,599
Macaca mulatta	MMUL 1.0	ENSMMUG00000007675	Chromosome 14: 73,742,253-73,752,788
Macropus eugenii	Meug_1.0 (GCA_000004035.1)	ENSMEUG00000008709	GeneScaffold_5800: 3,692-18,612
Meleagris gallopavo	UMD2 (GCA_000146605.1)	ENSMGAG00000015291	Chromosome 1: 203,021,087-203,026,274
Microcebus murinus	micMur1	ENSMICG00000015249	GeneScaffold_2542: 23,359-28,326
Monodelphis domestica	monDom5 (GCF_000002295.2)	ENSMODG00000006814	Chromosome 4: 342,820,867-342,826,459
Mus musculus	GRCm38 (GCA_000001635.3)	ENSMUSG00000070436	Chromosome 7: 99,345,375-99,353,230
Mustela putorius furo	MusPutFur1.0 (GCA_000215625.1)	ENSMPUG00000009062	Scaffold GL897030.1: 1,507,718-1,515,757
Myotis lucifugus	Myoluc2.0 (GCA_000147115.1)	ENSMLUG00000004105	Scaffold GL430204: 307,832-312,479
Nomascus leucogenys	Nleu1.0 (GCA_000146795.1)	ENSNLEG00000016882	SuperContig GL397264.1: 14,732,258-14,742,905
Ochotona princeps	OchPri2.0	ENSOPRG00000007518	GeneScaffold_3741: 324,406-328,787
Oreochromis niloticus	Orenil1.0 (GCA_000188235.1)	ENSONIG00000005220	Scaffold GL831147.1: 2,758,965-2,761,367
Oreochromis niloticus	Orenil1.0 (GCA_000188235.1)	ENSONIG00000015878	Scaffold GL831214.1: 2,676,278-2,680,779
Oreochromis niloticus	Orenil1.0 (GCA_000188235.1)	ENSONIG00000019907	Scaffold GL831388.1: 444,615-464,598
Oryctolagus cuniculus	OryCun2.0 (GCA_000003625.1)	ENSOCUG00000026029	Chromosome 1: 141,174,524-141,175,285
Oryzias latipes	MEDAKA1	ENSORLG00000014312	Chromosome 13: 27,993,919-27,996,591
Oryzias latipes	MEDAKA1	ENSORLT00000004598	Chromosome 18: 5,866,958-5,900,935
Otolemur garnettii	OtoGar3 (GCA_000181295.3)	ENSOGAG00000032244	Scaffold GL873520.1: 26,857,182-26,863,215
Ovis aries	Oar_v3.1 (GCA_000298735.1)	ENSOARG00000011824	Chromosome 15: 52,925,655-52,931,691
Pan troglodytes	CHIMP2.1.4 (GCA_000001515.4)	ENSPTRG00000004083	Chromosome 11: 73,325,614-73,336,379
Papio anubis	PapAnu2.0 (GCA_000264685.1)	ENSPANG00000015277	Chromosome 14: 65,410,443-65,424,007
Pelodiscus sinensis	PelSin_1.0 (GCA_000230535.1)	ENSPSIG00000008216	Scaffold JH210954.1: 315,916-321,989
Petromyzon Marinus	Pmarinus_7.0 (GCA_000148955.1)	ENSPMAG00000007485	Scaffold GL481971: 179,843-181,907
Poecilia formosa	Poecilia_formosa-5.1.2 (GCA_000485575.1)	ENSPFOG00000005984	Scaffold KI519751.1: 1,375,174-1,379,276
Poecilia formosa	Poecilia_formosa-5.1.2 (GCA_000485575.1)	ENSPFOG00000001716	Scaffold KI520426.1: 60,059-64,596
Poecilia formosa	Poecilia_formosa-5.1.2 (GCA_000485575.1)	ENSPFOG00000001241	Scaffold KI519773.1: 208,366-229,202

Table S2. Summary of 15 genes flanking on the each side of Human HSP47.

Gene	Description	Chromosomal Location	Strand
KCNE3	potassium channel, voltage gated subfamily E regulatory beta subunit 3	11 : 74,454,841 - 74,467,729	minus
LIPT2	lipoyl(octanoyl) transferase 2 (putative)	11 : 74,491,712 - 74,493,733	minus
POLD3	polymerase (DNA-directed), delta 3, accessory subunit	11 : 74,493,851 - 74,669,117	plus
CHRDL2	chordin-like 2	11 : 74,696,429 - 74,731,385	minus
RNF169	ring finger protein 169	11 : 74,748,868 - 74,842,413	plus
XRRA1	X-ray radiation resistance associated 1	11 : 74,807,739 - 74,949,200	minus
SPCS2	signal peptidase complex subunit 2 homolog (S. cerevisiae)	11 : 74,949,247 - 74,979,031	plus
NEU3	sialidase 3 (membrane sialidase)	11 : 74,988,134 - 75,018,893	plus
OR2AT4	olfactory receptor, family 2, subfamily AT, member 4	11 : 75,088,713 - 75,089,754	minus
SLCO2B1	solute carrier organic anion transporter family, member 2B1	11 : 75,100,563 - 75,206,549	plus
TPBGL	trophoblast glycoprotein-like	11 : 75,240,905 - 75,243,697	plus
ARRB1	*arrestin, beta 1*	*11 : 75,264,182 - 75,351,705*	*minus*
RPS3	*ribosomal protein S3*	*11 : 75,399,486 - 75,422,280*	*plus*
KLHL35	*kelch-like family member 35*	*11 : 75,422,394 - 75,430,629*	*minus*
GDPD5	*glycerophosphodiester phosphodiesterase domain containing 5*	*11 : 75,434,640 - 75,525,903*	*minus*
HSP47	***Heat Shock protein 47 kDA, HSP47, SERPINH1***	***11 : 75,562,056 - 75,572,783***	***plus***
MAP6	*microtubule-associated protein 6*	*11 : 75,586,918 - 75,669,120*	*minus*
MOGAT2	*monoacylglycerol O-acyltransferase 2*	*11 : 75,717,819 - 75,732,958*	*plus*
DGAT2	*diacylglycerol O-acyltransferase 2*	*11 : 75,759,512 - 75,801,535*	*plus*
UVRAG	*UV radiation resistance associated*	*11 : 75,815,167 - 76,143,195*	*plus*
WNT11	*wingless-type MMTV integration site family, member 11*	*11 : 76,186,325 - 76,210,736*	*minus*
PRKRIR	*protein-kinase, interferon-inducible double stranded RNA dependent inhibitor, repressor of (P58 repressor)*	*11 : 76,349,956 - 76,380,971*	*minus*
RP11-111M22.2	RP11-111M22.2	11 : 76,381,313 - 76,414,619	plus
C11orf30	chromosome 11 open reading frame 30	11 : 76,444,923 - 76,553,025	plus
LRRC32	*leucine rich repeat containing 32*	*11: 76,657,524 - 76,670,747*	*minus*

TSKU	*tsukushi, small leucine rich proteoglycan*	*11 : 76,782,251 - 76,798,154*	*plus*
ACER3	*alkaline ceramidase 3*	*11 : 76,860,867 - 77,026,797*	*plus*
B3GNT6	*UDP-GlcNAc:betaGal beta-1,3-N-acetylglucosaminyltransferase 6*	*11 : 77,034,398 - 77,041,973*	*plus*
CAPN5	*calpain 5*	*11 : 77,066,932 - 77,126,155*	*plus*
OMP	*olfactory marker protein*	*11 : 77,102,840 - 77,103,331*	*plus*
MYO7A	*myosin VIIA*	*11 : 77,128,264 - 77,215,239*	*plus*

Table S3. Detailed overview of gene annotation for the flanking genes on the ancestral locus of HSP47/SERPINH1 on the scaffold00131 from Japanese lamprey genome, A total 45 genes are residing on this locus of size 1 Mb. The gene g32.t1 is LjaHSP47/SERPINH1 and the g19.t1 is P2RY6-like GPCR (also known as lysophosphatic acid receptor, LPA6R) and these two genes are conserved in several vertebrate genomes (**Fig. 3**) and hence marked in red color. Gene annotation was performed using BLAST2GO 3.0 [8] and INTERPRO (www.ebi.ac.uk/**interpro**).

Gene ID	Gene Annotation	Protein Length	e-Value	sim mean	#GO	GO Names list	Enzyme Codes list	InterPro IDs
**g1.t1**	diacylglycerol kinase partial	99	5,40E-57	96%	22	C:cytosol; C:cytoskeleton; C:plasma membrane; C:cytoplasmic membrane-bounded vesicle; C:nuclear speck; F:diacylglycerol kinase activity; F:ATP binding; F:kinase binding; F:activating transcription factor binding; F:protein homodimerization activity; F:phospholipase binding; F:metal ion binding; F:protein heterodimerization activity; P:regulation of transcription from RNA polymerase II promoter; P:protein kinase C-activating G-protein coupled receptor signaling pathway; P:phosphorylation; P:cAMP-mediated signaling; P:response to ATP; P:diacylglycerol metabolic process; P:protein	EC:2.7.1.10 7	IPR001206 (SMART); IPR001206 (PFAM); PTHR11255 (PANTHER); PTHR11255:SF30 (PANTHER); IPR001206 (PROSITE_PROFILES); IPR016064 (SUPERFAMILY)

						homooligomerization; P:thrombin receptor signaling pathway; P:protein kinase C signaling		
**g2.t1**	---NA---	109	-	-		-	-	CYTOPLASMIC_DOMAIN (PHOBIUS); NON_CYTOPLASMIC_DOMAIN (PHOBIUS); TRANSMEMBRANE (PHOBIUS); TRANSMEMBRANE (PHOBIUS); CYTOPLASMIC_DOMAIN (PHOBIUS); NON_CYTOPLASMIC_DOMAIN (PHOBIUS); CYTOPLASMIC_DOMAIN (PHOBIUS); TRANSMEMBRANE (PHOBIUS); TRANSMEMBRANE (PHOBIUS); TMhelix (TMHMM); TMhelix (TMHMM); TMhelix (TMHMM); TMhelix (TMHMM)
**g3.t1**	---NA---	108	-	-		-	-	Coil (COILS)
**g4.t1**	phosphatidylinositol -glycan biosynthesis class f protein	207	2,10E-33	76,05 %	5	C:endoplasmic reticulum membrane; C:integral component of membrane; F:ethanolaminephosphotransferase activity; P:C-terminal protein lipidation; P:preassembly of GPI anchor in ER membrane	EC:2.7.8.1	IPR009580 (PFAM); IPR009580 (PANTHER); SIGNAL_PEPTIDE (PHOBIUS); TRANSMEMBRANE (PHOBIUS); NON_CYTOPLASMIC_DOMAIN (PHOBIUS); CYTOPLASMIC_DOMAIN (PHOBIUS); CYTOPLASMIC_DOMAIN (PHOBIUS); TRANSMEMBRANE

								(PHOBIUS); SIGNAL_PEPTIDE_N_REGION (PHOBIUS); NON_CYTOPLASMIC_DOMAIN (PHOBIUS); SIGNAL_PEPTIDE_H_REGION (PHOBIUS); TRANSMEMBRANE (PHOBIUS); SIGNAL_PEPTIDE_C_REGION (PHOBIUS); TMhelix (TMHMM); TMhelix (TMHMM); TMhelix (TMHMM); TMhelix (TMHMM)
**g5.t1**	ovotransferrin-like	794	0,00E+0 0	58,80 %	7	C:extracellular space; C:intracellular; F:GTP binding; F:metal ion binding; P:ion transport; P:small GTPase mediated signal transduction; P:iron ion homeostasis	-	IPR001156 (PRINTS); IPR001156 (SMART); IPR001156 (PFAM); G3DSA:3.40.190.10 (GENE3D); G3DSA:3.40.190.10 (GENE3D); IPR016357 (PIRSF); G3DSA:3.40.190.10 (GENE3D); G3DSA:3.40.190.10 (GENE3D); PTHR11485 (PANTHER); IPR018195 (PROSITE_PATTERNS); IPR018195 (PROSITE_PATTERNS); IPR001156 (PROSITE_PROFILES); IPR001156 (PROSITE_PROFILES); SSF53850 (SUPERFAMILY); SSF53850 (SUPERFAMILY)
**g6.t1**	conserved oligomeric golgi complex subunit 3	913	0,00E+0 0	74,25 %	13	C:nucleoplasm; C:cis-Golgi network; C:plasma membrane; C:Golgi transport complex; F:protein transporter activity; P:retrograde	-	-

						transport, vesicle recycling within Golgi; P:protein glycosylation; P:intracellular protein transport; P:ER to Golgi vesicle-mediated transport; P:retrograde vesicle-mediated transport, Golgi to ER; P:Golgi organization; P:protein localization to organelle; P:protein stabilization		
**g7.t1**	hypothetical chloroplast rf2	813	2,70E-18	50,30 %	6	C:microtubule; C:chloroplast stroma; F:calmodulin binding; F:ATP binding; F:microtubule binding; P:microtubule cytoskeleton organization	-	Coil (COILS); IPR029281 (PFAM); TRANSMEMBRANE (PHOBIUS); NON_CYTOPLASMIC_DOMAIN (PHOBIUS); TRANSMEMBRANE (PHOBIUS); TRANSMEMBRANE (PHOBIUS); TRANSMEMBRANE (PHOBIUS); CYTOPLASMIC_DOMAIN (PHOBIUS); CYTOPLASMIC_DOMAIN (PHOBIUS); CYTOPLASMIC_DOMAIN (PHOBIUS); NON_CYTOPLASMIC_DOMAIN (PHOBIUS); IPR011044 (SUPERFAMILY); SSF58113 (SUPERFAMILY); TMhelix (TMHMM); TMhelix (TMHMM); TMhelix (TMHMM)
**g8.t1**	glypican- partial	202	3,80E-07	68,55 %	4	C:proteinaceous extracellular matrix; C:plasma membrane;	-	IPR001863 (PFAM); IPR001863 (PANTHER); IPR031188

						C:anchored component of membrane; F:heparan sulfate proteoglycan binding		(PTHR10822:PANTHER)
**g9.t1**	glypican-5 isoform x25	537	1,40E-101	60,35 %	4	C:proteinaceous extracellular matrix; C:plasma membrane; C:anchored component of membrane; F:heparan sulfate proteoglycan binding	-	IPR001863 (PFAM); IPR031188 (PTHR10822:PANTHER); IPR001863 (PANTHER); IPR001863 (PANTHER)
**g10.t1**	endoplasmic reticulum-golgi intermediate compartment protein partial	161	1,20E-86	88,15 %	5	C:endoplasmic reticulum membrane; C:Golgi apparatus; C:integral component of membrane; C:endoplasmic reticulum-Golgi intermediate compartment membrane; P:vesicle-mediated transport	-	IPR012936 (PFAM); PTHR10984 (PANTHER); NON_CYTOPLASMIC_DOMAIN (PHOBIUS); TRANSMEMBRANE (PHOBIUS); CYTOPLASMIC_DOMAIN (PHOBIUS); TMhelix (TMHMM)
**g11.t1**	endoplasmic reticulum-golgi intermediate compartment protein 3 isoform x1	266	2,50E-89	76,95 %	5	C:endoplasmic reticulum membrane; C:Golgi apparatus; C:integral component of membrane; C:endoplasmic reticulum-Golgi intermediate compartment membrane; P:vesicle-mediated transport	-	PF13850 (PFAM); IPR012936 (PFAM); PTHR10984 (PANTHER); PTHR10984:SF25 (PANTHER)
**g12.t1**	progestin and adipoq receptor family member 9	354	4,10E-74	59,30 %	2	C:integral component of membrane; F:receptor activity	-	IPR004254 (PFAM); PTHR20855:SF29 (PANTHER); PTHR20855:SF29 (PANTHER); IPR004254 (PANTHER); IPR004254 (PANTHER); TRANSMEMBRANE (PHOBIUS); TRANSMEMBRANE (PHOBIUS); NON_CYTOPLASMIC_DOMAIN

								(PHOBIUS); TRANSMEMBRANE (PHOBIUS); TRANSMEMBRANE (PHOBIUS); TRANSMEMBRANE (PHOBIUS); NON_CYTOPLASMIC_DOMAIN (PHOBIUS); NON_CYTOPLASMIC_DOMAIN (PHOBIUS); CYTOPLASMIC_DOMAIN (PHOBIUS); NON_CYTOPLASMIC_DOMAIN (PHOBIUS); TRANSMEMBRANE (PHOBIUS); CYTOPLASMIC_DOMAIN (PHOBIUS); TRANSMEMBRANE (PHOBIUS); CYTOPLASMIC_DOMAIN (PHOBIUS); TRANSMEMBRANE (PHOBIUS); CYTOPLASMIC_DOMAIN (PHOBIUS); CYTOPLASMIC_DOMAIN (PHOBIUS); TMhelix (TMHMM); TMhelix (TMHMM); TMhelix (TMHMM); TMhelix (TMHMM); TMhelix (TMHMM); TMhelix (TMHMM); TMhelix (TMHMM); TMhelix (TMHMM)
**g13.t 1**	procollagen c-endopeptidase	389	3,10E-64	52,75 %	5	C:extracellular exosome; F:collagen binding; F:heparin binding;	-	IPR000859 (SMART); G3DSA:2.40.50.120 (GENE3D);

	enhancer 2					F:peptidase activator activity; P:positive regulation of peptidase activity		IPR000859 (G3DSA:2.60.120.GENE3D); IPR000859 (PFAM); IPR000859 (G3DSA:2.60.120.GENE3D); PTHR10127:SF40 (PANTHER); PTHR10127 (PANTHER); PTHR10127:SF40 (PANTHER); PTHR10127 (PANTHER); NON_CYTOPLASMIC_DOMAIN (PHOBIUS); SIGNAL_PEPTIDE_N_REGION (PHOBIUS); SIGNAL_PEPTIDE_C_REGION (PHOBIUS); SIGNAL_PEPTIDE (PHOBIUS); SIGNAL_PEPTIDE_H_REGION (PHOBIUS); IPR000859 (PROSITE_PROFILES); IPR000859 (PROSITE_PROFILES); SignalP-TM (SIGNALP_EUK); IPR008993 (SUPERFAMILY); IPR000859 (SUPERFAMILY); IPR000859 (SUPERFAMILY); TMhelix (TMHMM)
**g14.t1**	short transient receptor potential channel 1	497	0,00E+00	84,20 %	16	C:cytosol; C:integral component of plasma membrane; C:basolateral plasma membrane; C:sarcomere; C:costamere; C:receptor complex; C:membrane raft; F:store-operated calcium channel activity; F:ion channel binding; F:inositol 1,4,5	-	IPR002153 (PRINTS); IPR005457 (PRINTS); IPR005821 (PFAM); IPR002153 (PANTHER); IPR005457 (PTHR10117:PANTHER); SIGNAL_PEPTIDE_H_REGION (PHOBIUS); TRANSMEMBRANE (PHOBIUS);

						trisphosphate binding; P:manganese ion transport; P:saliva secretion; P:positive regulation of release of sequestered calcium ion into cytosol; P:cytosolic calcium ion homeostasis; P:response to calcium ion; P:calcium ion transmembrane transport		NON_CYTOPLASMIC_DOMAIN (PHOBIUS); CYTOPLASMIC_DOMAIN (PHOBIUS); SIGNAL_PEPTIDE_C_REGION (PHOBIUS); NON_CYTOPLASMIC_DOMAIN (PHOBIUS); NON_CYTOPLASMIC_DOMAIN (PHOBIUS); TRANSMEMBRANE (PHOBIUS); TRANSMEMBRANE (PHOBIUS); TRANSMEMBRANE (PHOBIUS); TRANSMEMBRANE (PHOBIUS); TRANSMEMBRANE (PHOBIUS); NON_CYTOPLASMIC_DOMAIN (PHOBIUS); NON_CYTOPLASMIC_DOMAIN (PHOBIUS); CYTOPLASMIC_DOMAIN (PHOBIUS); SIGNAL_PEPTIDE_N_REGION (PHOBIUS); TRANSMEMBRANE (PHOBIUS); SIGNAL_PEPTIDE (PHOBIUS); CYTOPLASMIC_DOMAIN (PHOBIUS); CYTOPLASMIC_DOMAIN (PHOBIUS); TRANSMEMBRANE (PHOBIUS); SignalP-TM

								(SIGNALP_EUK); TMhelix (TMHMM); TMhelix (TMHMM); TMhelix (TMHMM); TMhelix (TMHMM); TMhelix (TMHMM); TMhelix (TMHMM); TMhelix (TMHMM)
**g15.t 1**	inhibitor of nuclear factor kappa-b kinase-interacting protein isoform x1	254	5,30E-04	47,44 %	2	C:endoplasmic reticulum membrane; C:integral component of membrane	-	Coil (COILS)
**g16.t 1**	ninein-like protein	366	4,70E-12	48,70 %	1	F:calcium ion binding	-	Coil (COILS); Coil (COILS); PTHR18905 (PANTHER)
**g17.t 1**	nucleoredoxin-like protein 2	128	2,00E-15	55,45 %	3	P:visual perception; P:sensory perception of smell; P:photoreceptor cell maintenance	-	IPR012336 (PFAM); IPR012336 (G3DSA:3.40.30.GENE3D); IPR029519 (PTHR13871:PANTHER); PTHR13871 (PANTHER); IPR012336 (SUPERFAMILY)
**g18.t 1**	---NA---	169	-	-		-	-	IPR029242 (PFAM); CYTOPLASMIC_DOMAIN (PHOBIUS); TRANSMEMBRANE (PHOBIUS); NON_CYTOPLASMIC_DOMAIN (PHOBIUS); TMhelix (TMHMM)
**g19.t 1**	**lysophosphatidic acid receptor 6, P2RY6-like GPCR**	**404**	**2,60E-54**	**57,85 %**	**4**	**C:plasma membrane; C:integral component of membrane; F:G-protein coupled receptor activity; P:G-protein coupled receptor signaling pathway**	**-**	**IPR023242 (PRINTS); IPR000276 (PFAM); IPR022533 (PFAM); G3DSA:1.20.1070.10 (GENE3D); PTHR24232 (PANTHER); IPR000276 (PROSITE_PATTERNS); TRANSMEMBRANE (PHOBIUS); NON_CYTOPLASMIC_DOMAIN**

								**(PHOBIUS); TRANSMEMBRANE (PHOBIUS); CYTOPLASMIC_DOMAIN (PHOBIUS); NON_CYTOPLASMIC_DOMAIN (PHOBIUS); CYTOPLASMIC_DOMAIN (PHOBIUS); TRANSMEMBRANE (PHOBIUS); TRANSMEMBRANE (PHOBIUS); TRANSMEMBRANE (PHOBIUS); TRANSMEMBRANE (PHOBIUS); CYTOPLASMIC_DOMAIN (PHOBIUS); NON_CYTOPLASMIC_DOMAIN (PHOBIUS); TRANSMEMBRANE (PHOBIUS); NON_CYTOPLASMIC_DOMAIN (PHOBIUS); CYTOPLASMIC_DOMAIN (PHOBIUS); IPR017452 (PROSITE_PROFILES); SSF81321 (SUPERFAMILY); TMhelix (TMHMM); TMhelix (TMHMM); TMhelix (TMHMM); TMhelix (TMHMM); TMhelix (TMHMM); TMhelix (TMHMM)**
**g20.t1**	ef-hand calcium-binding domain-containing protein 2	82	5,50E-26	85,10 %	1	F:calcium ion binding	-	IPR011992 (G3DSA:1.10.238.GENE3D); IPR011992 (PFAM); PTHR23050

								(PANTHER); IPR002048 (PROSITE_PROFILES); SSF47473 (SUPERFAMILY)
**g21.t 1**	---NA---	70	-	-		-	-	SIGNAL_PEPTIDE_N_REGION (PHOBIUS); SIGNAL_PEPTIDE_H_REGION (PHOBIUS); NON_CYTOPLASMIC_DOMAIN (PHOBIUS); SIGNAL_PEPTIDE (PHOBIUS); SIGNAL_PEPTIDE_C_REGION (PHOBIUS); SignalP-noTM (SIGNALP_EUK); SignalP-noTM (SIGNALP_GRAM_NEGATIVE); SignalP-TM (SIGNALP_GRAM_POSITIVE); TMhelix (TMHMM)
**g22.t 1**	coiled-coil domain-containing protein 160	326	7,20E-17	47,40 %	10	C:nucleoplasm; C:nucleolus; F:zinc ion binding; F:tubulin binding; F:histone binding; F:histone deacetylase binding; F:ribonucleoprotein complex binding; F:poly(A) RNA binding; F:phosphoprotein binding; F:scaffold protein binding	-	Coil (COILS); Coil (COILS); Coil (COILS); Coil (COILS)
**g23.t 1**	low quality protein: wd repeat-containing protein 78	665	0,00E+0 0	59,65 %	1	P:hematopoietic progenitor cell differentiation	-	IPR001680 (SMART); IPR015943 (G3DSA:2.130.10.GENE3D); IPR001680 (PFAM); PTHR12442 (PANTHER); PTHR12442 (PANTHER); PTHR12442:SF12

								(PANTHER); PTHR12442:SF12 (PANTHER); IPR001680 (PROSITE_PROFILES); IPR017986 (PROSITE_PROFILES); IPR017986 (SUPERFAMILY)
**g24.t 1**	growth hormone secretagogue receptor type 1	260	4,60E-54	67,65 %	21	C:cell surface; C:integral component of membrane; C:neuron projection; C:membrane raft; F:growth hormone secretagogue receptor activity; F:growth hormone-releasing hormone receptor activity; P:G-protein coupled receptor signaling pathway; P:actin polymerization or depolymerization; P:adult feeding behavior; P:hormone-mediated signaling pathway; P:growth hormone secretion; P:response to food; P:positive regulation of appetite; P:negative regulation of interleukin-1 beta production; P:cellular response to insulin stimulus; P:positive regulation of multicellular organism growth; P:negative regulation of tumor necrosis factor biosynthetic process; P:positive regulation of insulin-like growth factor receptor signaling pathway; P:negative regulation of interleukin-6	-	IPR000276 (PRINTS); IPR000276 (PFAM); G3DSA:1.20.1070.10 (GENE3D); PTHR24243 (PANTHER); PTHR24243:SF3 (PANTHER); TRANSMEMBRANE (PHOBIUS); CYTOPLASMIC_DOMAIN (PHOBIUS); SIGNAL_PEPTIDE_C_REGION (PHOBIUS); NON_CYTOPLASMIC_DOMAIN (PHOBIUS); TRANSMEMBRANE (PHOBIUS); CYTOPLASMIC_DOMAIN (PHOBIUS); SIGNAL_PEPTIDE (PHOBIUS); SIGNAL_PEPTIDE_H_REGION (PHOBIUS); TRANSMEMBRANE (PHOBIUS); NON_CYTOPLASMIC_DOMAIN (PHOBIUS); SIGNAL_PEPTIDE_N_REGION (PHOBIUS); IPR017452 (PROSITE_PROFILES); SSF81321 (SUPERFAMILY); TMhelix (TMHMM); TMhelix (TMHMM);

						biosynthetic process; P:negative regulation of inflammatory response; P:regulation of synapse assembly		TMhelix (TMHMM); TMhelix (TMHMM)
**g25.t 1**	fibronectin type iii domain-containing protein 3b	843	2,00E-152	50,75 %	10	C:endoplasmic reticulum; C:integral component of membrane; F:protein binding; F:poly(A) RNA binding; P:cell migration; P:substrate adhesion-dependent cell spreading; P:positive regulation of fat cell differentiation; P:negative regulation of osteoblast differentiation; P:positive regulation of fibroblast proliferation; P:Type II pneumocyte differentiation	-	Coil (COILS); IPR003961 (SMART); IPR013783 (G3DSA:2.60.40.GENE3D); IPR003961 (PFAM); IPR013783 (G3DSA:2.60.40.GENE3D); IPR013783 (G3DSA:2.60.40.GENE3D); PTHR19900 (PANTHER); PTHR19900 (PANTHER); PTHR19900:SF49 (PANTHER); PTHR19900:SF49 (PANTHER); NON_CYTOPLASMIC_DOMAIN (PHOBIUS); TRANSMEMBRANE (PHOBIUS); NON_CYTOPLASMIC_DOMAIN (PHOBIUS); CYTOPLASMIC_DOMAIN (PHOBIUS); TRANSMEMBRANE (PHOBIUS); IPR003961 (PROSITE_PROFILES); IPR003961 (PROSITE_PROFILES); IPR003961 (PROSITE_PROFILES); IPR003961 (PROSITE_PROFILES); IPR003961 (PROSITE_PROFILES); IPR003961 (PROSITE_PROFILES); IPR003961 (SUPERFAMILY); IPR003961

								(SUPERFAMILY); IPR003961 (SUPERFAMILY); IPR003961 (SUPERFAMILY); TMhelix (TMHMM); TMhelix (TMHMM)
**g26.t 1**	---NA---	438	-	-		-	-	no IPS match
**g27.t 1**	fibronectin type iii domain-containing protein 3b	94	4,80E-08	70,71 %		-	-	PTHR19900 (PANTHER); PTHR19900:SF54 (PANTHER)
**g28.t 1**	---NA---	201	-	-		-	-	TRANSMEMBRANE (PHOBIUS); NON_CYTOPLASMIC_DOMAIN (PHOBIUS); CYTOPLASMIC_DOMAIN (PHOBIUS); NON_CYTOPLASMIC_DOMAIN (PHOBIUS); CYTOPLASMIC_DOMAIN (PHOBIUS); TRANSMEMBRANE (PHOBIUS); TRANSMEMBRANE (PHOBIUS); TMhelix (TMHMM); TMhelix (TMHMM); TMhelix (TMHMM)
**g29.t 1**	---NA---	423	-	-		-	-	no IPS match
**g30.t 1**	glycerol kinase 5	532	9,10E-175	64,60 %	6	C:mitochondrion; F:glycerol kinase activity; P:glycerol metabolic process; P:triglyceride metabolic process; P:phosphorylation; P:glycerol-3-phosphate biosynthetic process	EC:2.7.1.30	G3DSA:3.30.420.40 (GENE3D); IPR018485 (PFAM); G3DSA:3.30.420.40 (GENE3D); IPR018484 (PFAM); PTHR10196:SF54 (PANTHER); PTHR10196 (PANTHER);

								PTHR10196 (PANTHER); PTHR10196:SF54 (PANTHER); SSF53067 (SUPERFAMILY); SSF53067 (SUPERFAMILY)
**g31.t 1**	zinc finger b-box domain-containing protein 1	236	1,70E-14	42,65 %	2	C:intracellular; F:zinc ion binding	-	Coil (COILS)
**g32.t 1**	**HSP47/SERPINH1**	**470**	**1,60E-105**	**68,05 %**	**14**	**C:collagen trimer; C:extracellular space; C:endoplasmic reticulum; C:endoplasmic reticulum-Golgi intermediate compartment; C:extracellular exosome; F:serine-type endopeptidase inhibitor activity; F:collagen binding; F:poly(A) RNA binding; F:unfolded protein binding; P:chondrocyte development involved in endochondral bone morphogenesis; P:negative regulation of endopeptidase activity; P:collagen fibril organization; P:collagen biosynthetic process; P:protein maturation**	**-**	**IPR023796 (SMART); G3DSA:3.30.497.10 (GENE3D); G3DSA:2.30.39.10 (GENE3D); IPR023796 (PFAM); IPR000215 (PANTHER); PTHR11461:SF27 (PANTHER); IPR023795 (PROSITE_PATTERNS); IPR023796 (SUPERFAMILY)**
**g33.t 1**	lysosome-associated membrane glycoprotein 1	380	3,30E-32	40,05 %	24	C:multivesicular body; C:integral component of plasma membrane; C:external side of plasma membrane; C:dendrite; C:platelet dense granule membrane; C:late endosome membrane;	-	IPR002000 (PFAM); IPR002000 (PANTHER); NON_CYTOPLASMIC_DOMAIN (PHOBIUS); CYTOPLASMIC_DOMAIN (PHOBIUS); TRANSMEMBRANE

						C:sarcolemma; C:melanosome; C:neuronal cell body; C:cytolytic granule; C:phagolysosome membrane; C:extracellular exosome; C:alveolar lamellar body; F:enzyme binding; F:protein domain specific binding; P:autophagy; P:granzyme-mediated apoptotic signaling pathway; P:viral process; P:positive regulation of natural killer cell degranulation; P:autophagic cell death; P:protein stabilization; P:establishment of protein localization to organelle; P:Golgi to lysosome transport; P:regulation of organelle transport along microtubule		(PHOBIUS); TRANSMEMBRANE (PHOBIUS); NON_CYTOPLASMIC_DOMAIN (PHOBIUS); IPR002000 (PROSITE_PROFILES); TMhelix (TMHMM); TMhelix (TMHMM)
**g34.t 1**	haus augmin-like complex subunit partial	355	8,10E-08	63,10 %	4	C:centrosome; C:HAUS complex; P:spindle assembly; P:centrosome organization	-	Coil (COILS); SIGNAL_PEPTIDE (PHOBIUS); SIGNAL_PEPTIDE_H_REGION (PHOBIUS); SIGNAL_PEPTIDE_N_REGION (PHOBIUS); NON_CYTOPLASMIC_DOMAIN (PHOBIUS); SIGNAL_PEPTIDE_C_REGION (PHOBIUS)
**g35.t 1**	---NA---	225	-	-		-	-	no IPS match
**g36.t**	adp-ribosylation	208	3,10E-53	67,15	3	C:intracellular; F:GTP binding;	-	IPR006689 (PRINTS); IPR024156

**1**	factor			%		P:small GTPase mediated signal transduction		(SMART); IPR003579 (SMART); IPR006687 (SMART); IPR027417 (G3DSA:3.40.50.GENE3D); IPR006689 (PFAM); IPR005225 (TIGRFAM); PTHR11711 (PANTHER); IPR024156 (PROSITE_PROFILES); IPR027417 (SUPERFAMILY)
**g37.t 1**	rcc1 and btb domain-containing protein 1	531	0,00E+0 0	83,15 %	1	C:cytoplasm	-	IPR000408 (PRINTS); IPR000210 (SMART); IPR009091 (G3DSA:2.130.10.GENE3D); IPR000210 (PFAM); IPR000408 (PFAM); G3DSA:3.30.710.10 (GENE3D); PTHR22870:SF133 (PANTHER); PTHR22870 (PANTHER); IPR000408 (PROSITE_PATTERNS); IPR000408 (PROSITE_PROFILES); IPR000408 (PROSITE_PROFILES); IPR000408 (PROSITE_PROFILES); IPR000210 (PROSITE_PROFILES); IPR000408 (PROSITE_PROFILES); IPR000408 (PROSITE_PROFILES); IPR009091 (SUPERFAMILY); IPR011333 (SUPERFAMILY)
**g38.t 1**	neuroligin-3 isoform x4	656	0,00E+0 0	66,25 %	6	C:integral component of plasma membrane; C:synapse; F:neurexin family protein binding; P:cell adhesion; P:regulation of synaptic transmission; P:synapse	-	IPR000460 (PRINTS); IPR029058 (G3DSA:3.40.50.GENE3D); IPR002018 (PFAM); IPR030025 (PTHR11559:PANTHER); IPR030025 (PTHR11559:PANTHER);

						organization		PTHR11559 (PANTHER); NON_CYTOPLASMIC_DOMAIN (PHOBIUS); CYTOPLASMIC_DOMAIN (PHOBIUS); TRANSMEMBRANE (PHOBIUS); IPR029058 (SUPERFAMILY); TMhelix (TMHMM)
**g39.t1**	neuroligin- x-linked-like	187	2,50E-69	70,55 %	42	C:extracellular space; C:integral component of plasma membrane; C:cell surface; C:postsynaptic density; C:cell junction; C:endocytic vesicle; C:postsynaptic membrane; C:excitatory synapse; C:inhibitory synapse; F:receptor activity; F:neurexin family protein binding; F:cell adhesion molecule binding; F:carboxylic ester hydrolase activity; F:scaffold protein binding; P:regulation of respiratory gaseous exchange by neurological system process; P:receptor-mediated endocytosis; P:neuron cell-cell adhesion; P:metabolic process; P:visual learning; P:sensory perception of pain; P:adult behavior; P:positive regulation of synaptic transmission, GABAergic; P:social behavior; P:locomotory exploration behavior; P:axon extension; P:oligodendrocyte	EC:3.1.1.1	IPR002018 (PFAM); IPR029058 (G3DSA:3.40.50.GENE3D); PTHR11559 (PANTHER); PTHR11559 (PANTHER); IPR030025 (PTHR11559:PANTHER); IPR029058 (SUPERFAMILY)

						differentiation; P:neuromuscular process controlling balance; P:positive regulation of synapse assembly; P:positive regulation of synaptic transmission, glutamatergic; P:rhythmic synaptic transmission; P:regulation of dendritic spine morphogenesis; P:vocalization behavior; P:postsynaptic membrane assembly; P:presynaptic membrane assembly; P:gephyrin clustering involved in postsynaptic density assembly; P:postsynaptic density protein 95 clustering; P:positive regulation of inhibitory postsynaptic membrane potential; P:regulation of long-term synaptic potentiation; P:regulation of N-methyl-D-aspartate selective glutamate receptor activity; P:regulation of terminal button organization; P:positive regulation of excitatory postsynaptic membrane potential; P:positive regulation of alpha-amino-3-hydroxy-5-methyl-4-isoxazole propionate selective glutamate receptor activity		
**g40.t**	neuroligin-2-like	164	2,50E-57	84,95	53	C:extracellular space; C:Golgi	EC:3.1.1.1	IPR029058

**1**	isoform x5			%		apparatus; C:external side of plasma membrane; C:N-methyl-D-aspartate selective glutamate receptor complex; C:cell junction; C:filopodium tip; C:dendritic spine; C:postsynaptic membrane; C:excitatory synapse; C:inhibitory synapse; C:neuronal postsynaptic density; F:receptor activity; F:PDZ domain binding; F:neurexin family protein binding; F:protein dimerization activity; F:cell adhesion molecule binding; F:carboxylic ester hydrolase activity; F:scaffold protein binding; P:regulation of respiratory gaseous exchange by neurological system process; P:protein targeting; P:heterophilic cell-cell adhesion via plasma membrane cell adhesion molecules; P:neuron cell-cell adhesion; P:metabolic process; P:positive regulation of circadian sleep/wake cycle, wakefulness; P:synaptic vesicle targeting; P:calcium-dependent cell-cell adhesion via plasma membrane cell adhesion molecules; P:neuronal signal transduction; P:neuron projection development; P:positive		(G3DSA:3.40.50.GENE3D); IPR002018 (PFAM); PTHR11559:SF176 (PANTHER); PTHR11559 (PANTHER); IPR019819 (PROSITE_PATTERNS); SIGNAL_PEPTIDE_C_REGION (PHOBIUS); NON_CYTOPLASMIC_DOMAIN (PHOBIUS); SIGNAL_PEPTIDE (PHOBIUS); SIGNAL_PEPTIDE_H_REGION (PHOBIUS); SIGNAL_PEPTIDE_N_REGION (PHOBIUS); SignalP-TM (SIGNALP_GRAM_POSITIVE); SignalP-noTM (SIGNALP_GRAM_NEGATIVE); SignalP-noTM (SIGNALP_EUK); IPR029058 (SUPERFAMILY)

						regulation of synaptic transmission, GABAergic; P:rhythmic process; P:cytoskeletal matrix organization at active zone; P:protein homooligomerization; P:protein heterotetramerization; P:positive regulation of filopodium assembly; P:positive regulation of synapse assembly; P:positive regulation of synaptic transmission, glutamatergic; P:positive regulation of dendritic spine development; P:negative regulation of dendritic spine morphogenesis; P:terminal button organization; P:synaptic vesicle clustering; P:postsynaptic membrane assembly; P:alpha-amino-3-hydroxy-5-methyl-4-isoxazole propionate selective glutamate receptor clustering; P:N-methyl-D-aspartate receptor clustering; P:neurexin clustering involved in presynaptic membrane assembly; P:postsynaptic density protein 95 clustering; P:receptor localization to synapse; P:positive regulation of ruffle assembly; P:positive regulation of synaptic vesicle endocytosis; P:positive regulation of intracellular signal		

						transduction; P:positive regulation of synaptic vesicle exocytosis; P:regulation of N-methyl-D-aspartate selective glutamate receptor activity; P:regulation of alpha-amino-3-hydroxy-5-methyl-4-isoxazole propionate selective glutamate receptor activity; P:positive regulation of excitatory postsynaptic membrane potential		
**g41.t 1**	---NA---	96	-	-		-	-	no IPS match
**g42.t 1**	protein ect2 isoform x1	368	1,40E-35	55,80 %	9	C:nucleus; C:cytoplasm; F:Rho guanyl-nucleotide exchange factor activity; F:GTPase activator activity; P:cell morphogenesis; P:cytokinesis; P:intracellular signal transduction; P:positive regulation of GTPase activity; P:positive regulation of neuron differentiation	-	Coil (COILS); IPR026817 (PANTHER)
**g43.t 1**	hypothetical protein H310_04227	72	7,30E-04	61%		-	-	no IPS match
**g44.t 1**	protein ect2 isoform x1	100 2	5,30E-101	73,35 %	27	C:cytoplasm; C:bicellular tight junction; C:midbody; C:nuclear membrane; C:cleavage furrow; C:centralspindlin complex; F:signal transducer activity; F:Rho guanyl-nucleotide exchange factor activity; F:GTPase activator activity; F:Rho GTPase binding; F:protein	-	IPR001357 (SMART); IPR000219 (SMART); IPR001357 (G3DSA:3.40.50.GENE3D); IPR000219 (G3DSA:1.20.900.GENE3D); IPR000219 (PFAM); IPR001357 (PFAM); IPR026817 (PANTHER); IPR001331 (PROSITE_PATTERNS);

						homodimerization activity; P:cell morphogenesis; P:cytokinesis; P:activation of protein kinase activity; P:positive regulation of cytokinesis; P:intracellular signal transduction; P:positive regulation of protein import into nucleus; P:positive regulation of apoptotic process; P:positive regulation of I-kappaB kinase/NF-kappaB signaling; P:positive regulation of GTPase activity; P:positive regulation of neuron differentiation; P:protein homooligomerization; P:regulation of attachment of spindle microtubules to kinetochore; P:cellular response to hydrogen peroxide; P:bicellular tight junction assembly; P:cellular response to calcium ion; P:cellular response to ionizing radiation		TRANSMEMBRANE (PHOBIUS); NON_CYTOPLASMIC_DOMAIN (PHOBIUS); CYTOPLASMIC_DOMAIN (PHOBIUS); IPR001357 (PROSITE_PROFILES); IPR000219 (PROSITE_PROFILES); IPR001357 (SUPERFAMILY); IPR000219 (SUPERFAMILY); IPR001357 (SUPERFAMILY)
**g45.t1**	gpalpp motifs-containing protein 1	320	7,90E-56	55,25 %	3	F:molecular_function; P:biological_process; C:cellular_component	-	IPR022226 (PFAM); PTHR15606:SF3 (PANTHER); PTHR15606 (PANTHER)

red - matching to syntenic data in **Fig. 3**
